# Structural and functional dissection reveals distinct roles of Ca^2+^-binding sites in the giant adhesin SiiE of *Salmonella enterica*

**DOI:** 10.1371/journal.ppat.1006418

**Published:** 2017-05-30

**Authors:** Britta Peters, Johanna Stein, Stefan Klingl, Nathalie Sander, Achim Sandmann, Nicola Taccardi, Heinrich Sticht, Roman G. Gerlach, Yves A. Muller, Michael Hensel

**Affiliations:** 1 Abt. Mikrobiologie, Universität Osnabrück, Osnabrück, Germany; 2 Lehrstuhl für Biotechnik, FAU Erlangen-Nürnberg, Erlangen, Germany; 3 Institut für Biochemie, FAU Erlangen-Nürnberg, Erlangen, Germany; 4 Lehrstuhl für Chemische Reaktionstechnik, FAU Erlangen-Nürnberg, Erlangen, Germany; 5 Robert-Koch-Institut, Wernigerode, Germany; University of California Davis School of Medicine, UNITED STATES

## Abstract

The giant non-fimbrial adhesin SiiE of *Salmonella enterica* mediates the first contact to the apical site of epithelial cells and enables subsequent invasion. SiiE is a 595 kDa protein composed of 53 repetitive bacterial immunoglobulin (BIg) domains and the only known substrate of the SPI4-encoded type 1 secretion system (T1SS). The crystal structure of BIg50-52 of SiiE revealed two distinct Ca^2+^-binding sites per BIg domain formed by conserved aspartate or glutamate residues. In a mutational analysis Ca^2+^-binding sites were disrupted by aspartate to serine exchange at various positions in the BIg domains of SiiE. Amounts of secreted SiiE diminish with a decreasing number of intact Ca^2+^-binding sites. BIg domains of SiiE contain distinct Ca^2+^-binding sites, with type I sites being similar to other T1SS-secreted proteins and type II sites newly identified in SiiE. We functionally and structurally dissected the roles of type I and type II Ca^2+^-binding sites in SiiE, as well as the importance of Ca^2+^-binding sites in various positions of SiiE. Type I Ca^2+^-binding sites were critical for efficient secretion of SiiE and a decreasing number of type I sites correlated with reduced secretion. Type II sites were less important for secretion, stability and surface expression of SiiE, however integrity of type II sites in the C-terminal portion was required for the function of SiiE in mediating adhesion and invasion.

## Author summary

The interaction of *Salmonella enterica* with polarized epithelial cells depends on the function of SiiE, a 595 kDa adhesin containing 53 repeats of a bacterial immunoglobulin (BIg) domain. SiiE is secreted and surface-expressed by a cognate type I secretion system (T1SS). We found that BIg domains contain amino acid (aa) residues forming binding sites for Ca^2+^ ions. Two types of Ca^2+^-binding sites can be distinguished, termed type I and type II sites. We performed a structural and functional dissection of Ca^2+^-binding sites of SiiE. After mutation of aa residues forming type I and/or type II Ca^2+^-binding sites, we investigated the secretion, surface expression and function as adhesin for interaction with polarized epithelial cells of the SiiE variants. We found that Ca^2+^-binding sites are critical for supporting the secretion of SiiE. Integrity of type I sites in any position of SiiE is essential for efficient secretion and surface expression. In contrast integrity of type II sites is less important for secretion. However, loss of type II in the C-terminal, most distal portion of SiiE ablated SiiE-mediated adhesion, while loss of the type II sites in middle or N-terminal portions of SiiE had less or no effect on SiiE function. We propose a novel mechanism of Ca^2+^-dependent secretion and conformational fine tuning of SiiE as a large T1SS substrate with a central role in the interaction of *S*. *enterica* with host cells.

## Introduction

*Salmonella enterica* is a food-borne Gram-negative pathogen which causes self-limiting gastroenteritis. To survive inside the host, *Salmonella* possesses sophisticated virulence factors and protein secretion systems [1]. A *Salmonella* pathogenicity island (SPI) 1-encoded type 3 secretion system (T3SS) is necessary for invasion [2]. This protein secretion system is capable to secrete a distinct cocktail of effector proteins, which manipulate the host cell. In order to establish the initial contact to the apical side of polarized epithelial cells and to enable translocation by the SPI1-T3SS, *Salmonella* deploys the SPI4-encoded T1SS and the giant non-fimbrial adhesin SiiE [3].

The SPI4 locus encodes SiiE and the T1SS for secretion of SiiE, with SiiF being the inner membrane transport ATPase, SiiD acting as periplasmic adaptor protein (PAP), and outer membrane secretin SiiC [4]. SiiE, a 595 kDa non-fimbrial adhesin, is the only known substrate for the SPI4-T1SS [4]. SiiE mediates the first intimate contact to the host cell through binding to glycostructures containing N-acetyl-glucosamine and/or α2,3-linked sialic acid [5]. This contact positions the SPI1-T3SS to efficiently translocate effector proteins which lead to actin remodeling and macropinocytosis of the bacteria. As a T1SS substrate protein, SiiE possesses a C-terminal secretion signal [4]. The adhesin is transiently retained within the secretion system and at later time points present in the supernatant [6]. The two accessory proteins SiiA and SiiB are located in the inner membrane presumably forming a proton-conductive channel. This channel may use the proton motive force (PMF) at the cytoplasmic membrane to regulate the retention of SiiE, either through sensing the physiological state of the cell or by inducing conformational changes to binding partners [7].

The adhesin SiiE is composed of an N-terminal domain containing β-sheet and coiled-coil repeats, followed by 53 repeats of bacterial immunoglobulin (BIg) domains [6]. BIg52 and BIg53 are separated by a putatively unfolded element termed insertion. Sequence alignments of all 53 BIg domains revealed that a prototypical BIg domain possesses 6 conserved aspartate (D) or glutamate (E) residues, of which 5 form two binding sites for Ca^2+^ ions. Recently we solved the crystal structure of SiiE BIg50-52. Despite not having explicitly added any Ca^2+^ ions during protein production, the crystal structure revealed that up to two Ca^2+^ ions are bound per BIg domain in SiiE [8]. The SiiE-wide conservation of D and E residues that are involved in Ca^2+^ binding suggests that SiiE binds about 100 Ca^2+^ ions per molecule [8]. Ultrastructural analysis showed that chelation of Ca^2+^ ions of purified secreted SiiE molecules distorts the linear rod-like structure of SiiE, indicating a stabilizing effect of Ca^2+^ ions. Ca^2+^ binding has been demonstrated for other T1SS substrate proteins, such as adhesins, the antifreeze protein of *Marinomonas primoryensis* (*Mp*AFP) [9] or SpaA of *Corynebacterium diphtheria* [10]. Repeat in Toxin (RTX) proteins are a family of T1SS-secreted toxins and *E*. *coli* HlyA und *Bordetella pertussis* adenylate cyclase CyaA are well studied RTX toxins. The RTX motif is a glycine-rich nonapeptide involved in Ca^2+^ binding. For both HlyA and CyaA, binding of Ca^2+^ ions was shown to support the secretion by the T1SS [11–14].

The BIg domains of SiiE possess two distinct types of Ca^2+^-binding sites that are distinct from the RTX motif. The conserved D residues are numbered according to the multi-sequence alignment of SiiE BIg domains shown by Griessl *et al*. [8]. Type I Ca^2+^-binding sites are positioned at the interface of two BIg domains and contain three D residues, namely BIg_n-1_^117^D and BIg_n_^43^D and ^97^D. Type I Ca^2+^-binding sites are frequently found in BIg domain proteins. In contrast, type II Ca^2+^-binding sites are specific to SiiE and built by two D residues within one BIg domain (BIg_n_^16^D and ^24^D). Please note that each Ca^2+^ ion is coordinated by 6 ligands and therefore other residues and water molecules are involved as well in Ca^2+^ ion binding, in addition to the conserved D residues [8]. We also observed conserved tryptophan residues, i.e. ^74^W, in most of the BIg domains. This residue is distal to the Ca^2+^-binding sites, but may be involved in interaction of SiiE with glycostructures as observed for transport proteins [15] or fimbrial adhesins [16].

The roles of the distinct type I and type II Ca^2+^-binding sites for secretion of SiiE and function as adhesin are not known. Here, we report the functional dissection of SiiE Ca^2+^-binding sites. We found that with an increasing number of conserved D residues exchanged to the non-charged amino acid serine (S), the amounts of secreted SiiE were dramatically decreased. Exchanges of single D residues or of either single type I or type II Ca^2+^-binding sites showed no effect, while exchange of multiple type I or type II Ca^2+^-binding sites showed a more dramatic effect when type I Ca^2+^-binding sites are missing. Our data demonstrate a critical role of type I sites to support transport of SiiE through the T1SS, while type II sites are important to structure secreted SiiE and to maintain a BIg domain conformation that enables interaction with cognate ligands on the host cell surface.

## Results

### Exchanges of conserved aspartate residues in the C- and N-terminal moieties of SiiE affect amounts of secreted SiiE

**Fig 1:**
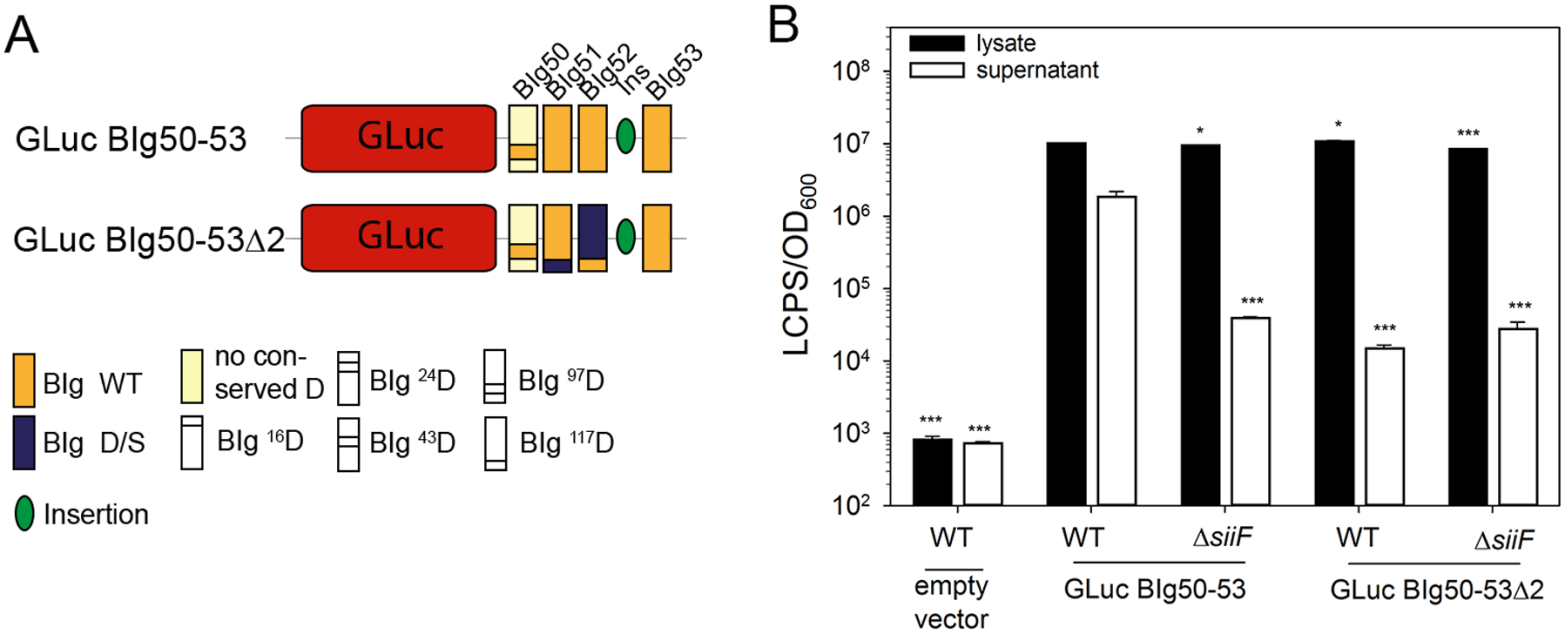
Role of Ca^2+^-binding sites for secretion of SiiE. A) Schematic overview of fusion proteins analyzed in this study. GLuc fused to SiiE contains BIg50–BIg53. Conserved aspartate residues exchanged to serine are shown in blue and the position of the residues is indicated in subscript. GLuc Big50-53Δ2 is a mutant form lacking two Ca^2+^-binding sites. B) GLuc assay for secretion of fusion proteins. *Salmonella* WT and Δ*siiF* strains harboring plasmids for synthesis of GLuc-SiiE fusions or empty vector were subcultured for 6 h. Samples were processed and GLuc activity determined as described in S1 Fig. Filled and open bars show total cell-associated GLuc activity (lysate) and secreted GLuc activity (supernatant), respectively. Experiments were performed in triplicates, one representative is shown. Statistical analysis was performed by one-way ANOVA with Bonferroni t-test and is indicated as follows: n.s., not significant; *, P < 0.05; **, P < 0.01; ***, P < 0.001.

The giant adhesin SiiE possesses 53 BIg domains, most of which contain five conserved D or E residues that coordinate binding of two Ca^2+^ ions. We investigated the role of Ca^2 +^-binding sites in BIg domains for secretion of SiiE. The *Gaussia* luciferase (GLuc) [17] was used as reporter for quantification of amounts of secreted SiiE (Fig 1, S1 Fig). Compared to Firefly luciferase, GLuc is ATP independent, and more robust and progressive [18]. To quantify the secretion, the reporter GLuc BIg50-53 was constructed by fusing GLuc to the C-terminal moiety of SiiE, i.e. BIg domains 50–53, the insertion and the C-terminal secretion signal (Fig 1A). Another construct was generated in which all five conserved D residues forming the type I and type II Ca^2+^-binding sites in BIg51 and BIg52 were exchanged to S (D/S exchange), termed GLuc BIg50-53Δ2. The number of deleted Ca^2+^-binding sites is indicated by Δn. A further reporter fusion consisting of GLuc and BIg47-53 was generated and D/S exchanges of various extent were introduced (S1C Fig).

The synthesis and secretion of GLuc fusions with WT or mutant SiiE portions was compared using *Salmonella* WT and the *siiF*-deficient strain unable to form a functional T1SS. GLuc activities in the lysate and supernatant obtained after 6 h of subculture represent the cytosolic and surface-bound, or secreted portion of GLuc fusions, respectively (S1 Fig, Fig 1B). GLuc activities in lysates were similar for GLuc BIg50-53 and GLuc BIg50-53Δ2 reporters, indicating similar rates of synthesis and stability (Fig 1B). Secreted GLuc activity for the SiiE WT reporter was 46.7-fold lower in the Δ*siiF* background, demonstrating SPI4-T1SS-dependent secretion of the reporter. In the SPI4-T1SS-proficient background, secreted GLuc activity for the GLuc BIg50-53Δ2 reporter was 122.4-fold lower than for the GLuc BIg50-53 reporter.

We also analyzed secretion of a GLuc fusion protein containing BIg47-53 (S1C and S1D Fig). Here, five D/S exchanges in GLuc BIg47-53Δ2 did not reduce secretion. Also, the exchanges resulting in GLuc BIg47-53Δ4 were without effect on the secretion of the reporter, while additional D/S exchanges for deletion of another two Ca^2+^-binding sites caused a five-fold reduced secretion of GLuc BIg47-53Δ6. The complete removal of 10 Ca^2+^-binding sites in GLuc BIg47-53Δ10 resulted in 73.7-fold reduced secretion, similar to levels of the WT reporter fusion in Δ*siiF* background.

We conclude that Ca^2+^-binding sites in BIg SiiE are required for the secretion of SiiE. The removal of two Ca^2+^-binding sites in a secretion reporter with four BIg was sufficient to ablate T1SS-dependent secretion, while removal of at least 6 Ca^2+^-binding sites was required to affect secretion of a reporter fusion with 7 BIg.

### The number of Ca^2+^-binding sites correlates with SiiE function

**Fig 2:**
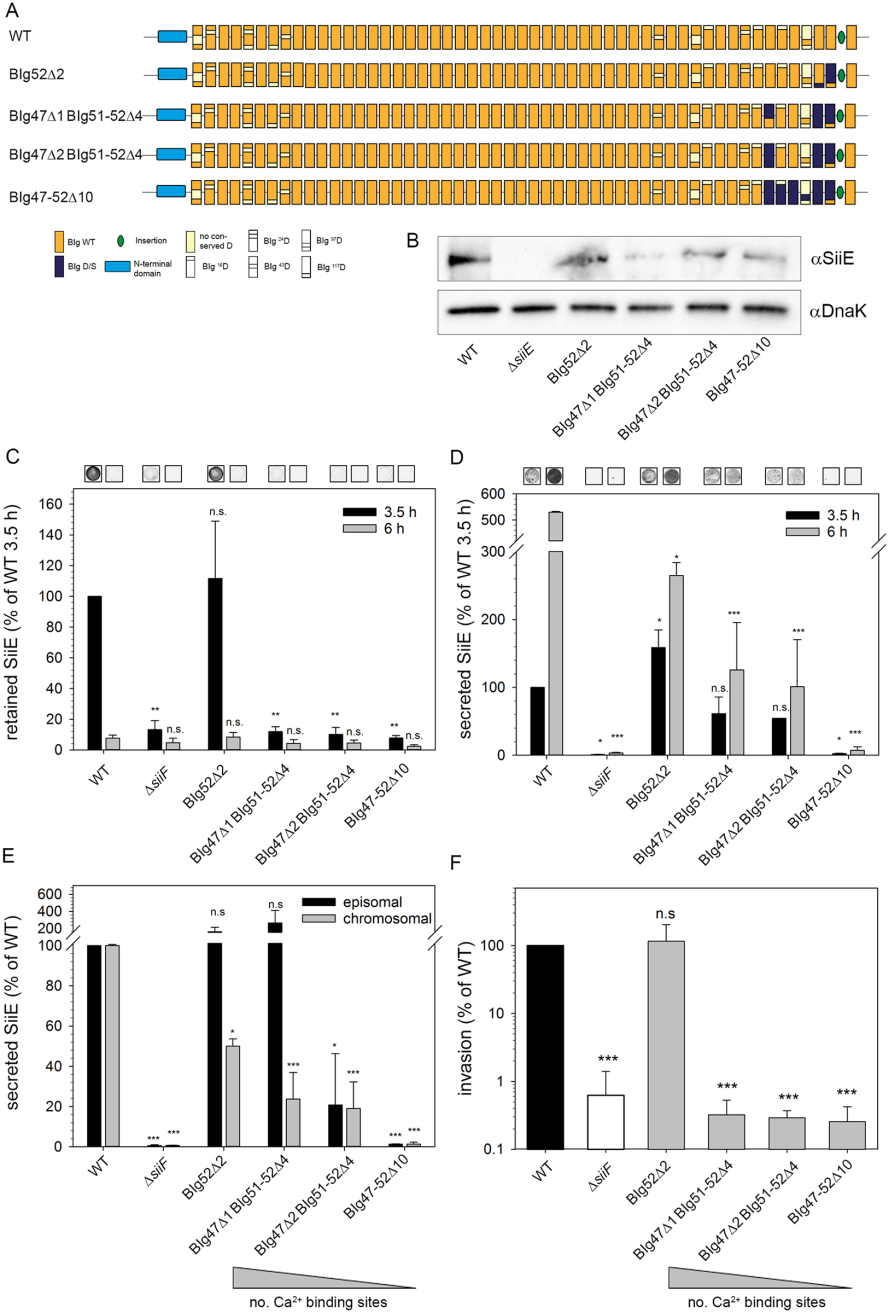
Role of Ca^2+^-binding sites in the C-terminal moiety for secretion and function of chromosomally encoded SiiE. A) Schematic overview of the domain organization of SiiE and chromosomally encoded forms of SiiE with mutated Ca^2+^-binding sites. The positions of deleted Ca^2+^-binding sites is shown in blue and Δ1, Δ2, Δ4 and Δ10 indicate 1, 2, 4, or 10 mutated Ca^2+^-binding sites, respectively. Mutant alleles were generated using the λ Red-mediated replacement of I-*Sce*I *aph* cassette by DNA fragments harboring sequence alterations. B) Western blot detection of SiiE in whole bacterial lysates of a 3.5 h subculture for analysis of protein synthesis. C) The amounts of SiiE retained on the bacterial surface of *Salmonella* WT and various mutant strains at 3.5 h and 6 h subculture was determined by dot blot analysis. Representative dot blots are shown. The loading of dot blots was normalized by parallel analysis of the signal for LPS. D) The amounts of secreted SiiE for *Salmonella* WT and various mutant strains at 3.5 h and 6 h subculture were determined by dot blot analysis. After TCA precipitation, the pellet was resuspended according to OD_600_ of the culture and equal amounts of samples were loaded onto a nitrocellulose membrane. E) Comparison of activities of secreted GLuc-SiiE reporters obtained for plasmid-encoded GLuc conjugated SiiE by GLuc assay (S1 Fig) or chromosomally encoded AA exchanges by dot blot analysis depicted in D). F) SiiE-dependent invasion of polarized epithelial MDCK cells by *Salmonella* WT, Δ*siiF*, and strains expressing various mutant alleles of *siiE*. Cells were infected with the indicated strains at a MOI of 5. Non-internalized bacteria were removed by washing and remaining bacteria were killed by addition of gentamicin for 1 h. Subsequently, cells were lysed and serial dilutions were plated onto agar plates for determination of colony-forming units (CFU). Invasion is depicted as percentage of the inoculum that was internalized by host cells. Experiments were performed in duplicates (C, D) or triplicates (E, F), and means and standard deviations are shown. Statistical analysis was performed by one-way ANOVA with Bonferroni t-test and is indicated as follows: n.s., not significant; *, P < 0.05; **, P < 0.01; ***, P < 0.001; ***, P < 0.001.

To analyze the role of Ca^2+^-binding sites in SiiE for SiiE-dependent virulence functions of *Salmonella*, we transferred mutant alleles with D/S exchanges of various extent into chromosomal *siiE* using λ Red recombineering (Fig 2A). The resulting strains synthesized mutant forms of SiiE with D/S exchanges resulting in removal of 2, 5, 6 or 10 Ca^2+^-binding sites (Fig 2B). No SiiE was detected for the Δ*siiE* strain and compared to WT SiiE, variable amounts of mutant SiiE were observed. Compared to the WT, all mutant strains investigated contain lower amount of cell-associated SiiE. Since whole bacterial lysates were analyzed, one cannot distinguish between SiiE present in cytosol, or SiiE retained on the bacterial surface. Mutant forms of SiiE with reduced surface retention will lead to lower amounts of cell-associated SiiE, although levels of SiiE synthesis are comparable to WT SiiE.

SiiE retention on the bacterial surface and secretion into culture supernatant was analyzed by dot blots of whole cells, and protein precipitated from culture supernatants, respectively (Fig 2C and 2D). Our previous work demonstrated that SiiE is mainly retained on the bacterial surface at 3.5 h of subculture, and predominantly released into the supernatant at 6 h and later of subculture [6]. Of the various mutant strains analyzed, only the strain producing SiiE BIg52Δ2 showed SiiE retention after 3.5 h of subculture similar to WT. After 6 h subculture levels of SiiE retention of WT and all mutant strains were as low as the negative control.

The number of mutated Ca^2+^-binding sites correlated with reduction of secreted SiiE at 3.5 and 6 h of subculture. With increasing numbers of D/S exchanges, lesser amounts of secreted SiiE were detected. Amounts of SiiE BIg47-52Δ10 were as low as the negative control, indicating a complete loss of secretion for this SiiE mutant (Fig 2D).

We compared secretion of WT and mutant SiiE quantified by dot blot analyses to GLuc activities of the GLuc-SiiE reporter (Fig 2E, S1C Fig). GLuc reporters for SiiE BIg52Δ2 and SiiE BIg47Δ1 BIg51-52Δ4 resulted in GLuc activities similar to GLuc-SiiE_WT_. If introduced in chromosomal *siiE*, the mutations resulted in reduced amounts of secreted SiiE. Secretion of SiiE BIg47Δ2 BIg51-52Δ4 was highly reduced in both assays, while no secretion of SiiE BIg47-52Δ10 was detected in GLuc and dot blot assays. The data demonstrate that Ca^2+^-binding sites in BIg are important for the secretion of SiiE, and that amounts of secreted SiiE decreases with an increasing number of D/S exchanges in BIg domains.

We next determined the effect of deletion of Ca^2+^-binding sites on SiiE-dependent virulence functions, i.e. adhesion to polarized epithelial cell followed by SPI1-T3SS-mediated invasion (Fig 2F). Only SiiE BIg52Δ2 conferred invasion of MDCK cells at a level comparable to WT SiiE. All other mutant SiiE we investigated resulted in highly reduced invasion, comparable to the *siiF*-deficient strain that is unable to secrete SiiE.

We conclude that Ca^2+^-binding sites in the C-terminal part of SiiE are essential for secretion and function of the adhesin. Removal of Ca^2+^-binding sites in more than one BIg in this moiety results in loss of function.

### The position of Ca^2+^-binding sites is critical for the function of SiiE

**Fig 3:**
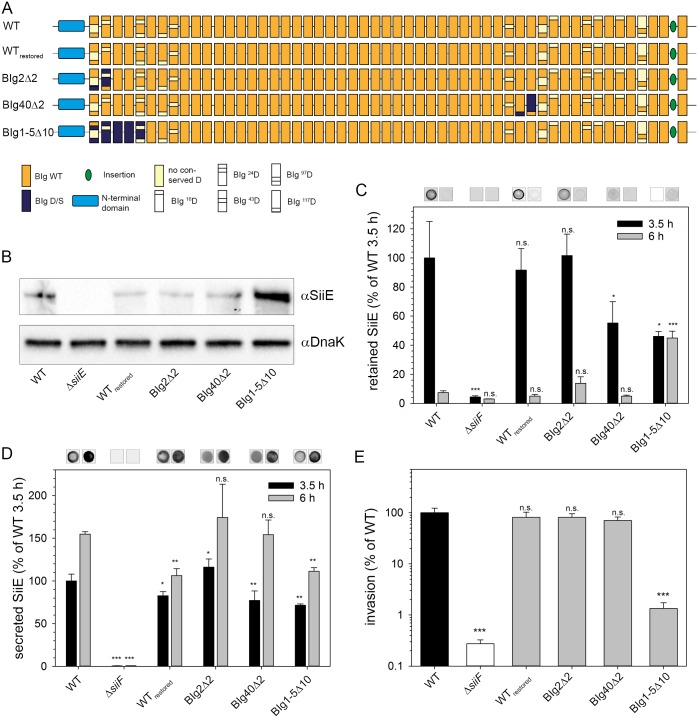
Role of Ca^2+^-binding sites in N-terminal or central portions of SiiE. A) Schematic overview of various mutant forms of SiiE with D/S exchanges in BIg2, BIg40 or BIg1-5 for deletion of 2 (Δ2) or 10 (Δ10) Ca^2+^-binding sites. B) Western blot for analyses of synthesis of mutant forms of SiiE. C) Amounts of retained SiiE after 3.5 h and 6 h of subculture. D) Secreted SiiE after 3.5 h and 6 h of subculture. E) SiiE-dependent invasion of polarized epithelial MDCK cells. Analyses of synthesis, surface retention and secretion and SiiE-dependent invasion of polarized cells were performed as described for Fig 2.

The C-terminal moiety of SiiE contains the signal for T1SS secretion, is secreted first and is likely to be exposed most distal to the cell envelope. We next tested the functional relevance of Ca^2+^-binding sites in the middle or N-terminal portions of SiiE. Strains were generated with mutations in chromosomal *siiE* resulting in deletion of Ca^2+^-binding sites in BIg2, BIg40, or BIg1-5 (Fig 3A). Since ^117^D of a previous BIg domain (BIg_(n-1)_) forms a type I Ca^2+^-binding site with ^43^D and ^97^D of a subsequent BIg domain (BIg_(n)_), ^117^D of BIg1 and BIg39 were exchanged instead of ^117^D of BIg2 and BIg40, resulting in SiiE BIg2Δ2 and SiiE BIg40Δ2, respectively. To control the precision of the Red recombineering method applied here and the absence of unwanted attenuating mutations, we used a *siiE* mutant strain and restored the WT sequence. This strain, termed WT_restored_, showed SiiE-dependent phenotypes as the WT strain.

Strains expressing mutant chromosomal *siiE* were tested for protein synthesis. No expression could be detected for the negative control Δ*siiE*. All mutant strains synthesized SiiE of correct size (Fig 3B). The deletion of Ca^2+^-binding sites only in BIg2 or BIg40 had no or only small effects on surface retention of SiiE (Fig 3C), secretion (Fig 3D), or SiiE-dependent invasion (Fig 3E). Secretion of SiiE BIg1-5Δ10 was slightly reduced, but there was still more secretion after 6 h of subculture than after 3.5 h of subculture. Interestingly, the level of SiiE retention was also reduced to approximately 50% of WT and maintained at the same level at 6 h of subculture. Destruction of all Ca^2+^-binding sites in BIg1-5 (BIg1-5Δ10) led to a 74.6-fold decreased invasion of polarized cells (Fig 3E), while the same extent of deletions in BIg47-52 (BIg47-52Δ10) resulted in 392.2-fold reduced invasion (Fig 2F). Compared to SiiE BIg47-52Δ10, reduction of retention and secretion is less pronounced for SiiE BIg1-5Δ10. If extracellular Ca^2+^ ions facilitate secretion of SiiE, the secretion might come to a halt earlier for BIg47-52Δ10 than for SiiE BIg1-5Δ10.

In addition to the conserved D or E residues involved in Ca^2+^ binding, 47 of 53 BIg domains possess a conserved tryptophan residue at position 74. To test a potential role of these conserved tryptophan residues in SiiE function, we performed W to F (W/F) exchanges in 1, 2, or 3 BIg in the C-terminal moiety of SiiE (S2A Fig). These mutations only resulted in minor changes of the amounts of SiiE retained and secreted at 3.5 h or 6 h of subculture (S2B, S2C and S2D Fig). Functionally, none of the mutant forms of SiiE with various degrees of W/F exchanges resulted in reduced invasion of polarized epithelial cells (S2E Fig), indicating that conserved ^74^W residues in the C-terminal moiety of SiiE are neither important for secretion and retention of SiiE, nor for the SiiE-dependent adhesion and invasion.

T1SS substrate proteins are secreted in an unfolded state [19]. For example, secretion of *E*. *coli* HlyA was highly reduced if the protein was modified in a way that allowed folding in the cytosol [20]. To further investigate parameters known to affect secretion of T1SS substrate proteins, we tested if folding rate influences secretion as for HlyA. We fused the C-terminal portion of SiiE harboring the secretion signal to MalE. Distinct point mutations in the MalE portion of the fusion protein led to different folding rates as previous established by Bakkes *et al*. [20]. Secretion of various MalE-SiiE fusion proteins was analyzed at 3.5 h and 6 h of subculture. Similar amounts of fusion proteins were detected in the culture supernatant at both time points (S3 Fig). This indicates that the rate of intracellular folding did not affect SiiE secretion, supporting that binding of extracellular Ca^2+^ ions by the secreted portion of BIg domains is more important.

### Molecular dynamics simulations for roles of type I and type II Ca^2+^-binding sites

**Fig 4:**
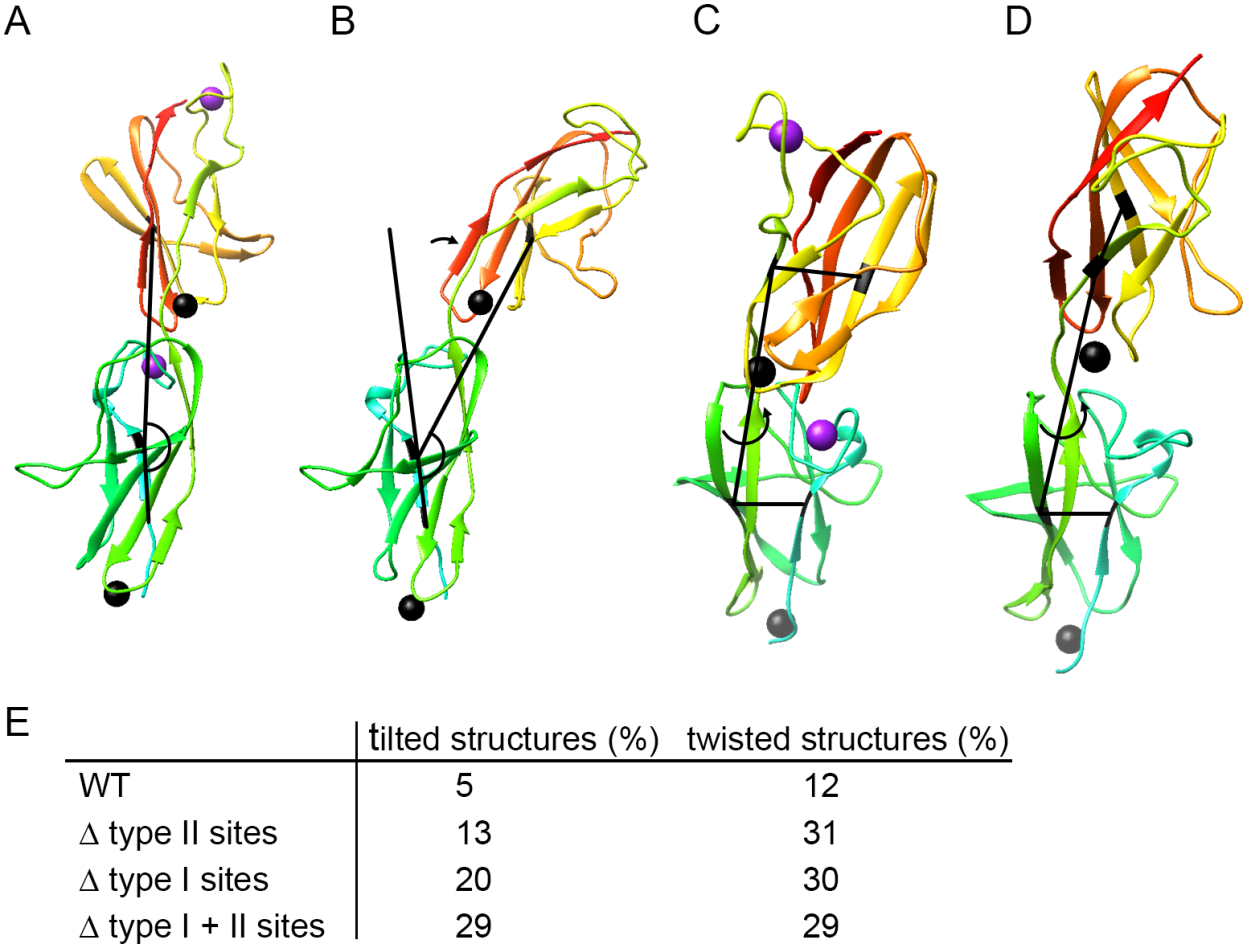
Effect of mutations of type I and II sites on the geometry of the BIg51-52 domain pair. Schematic presentation of the tilt and twist angles analyzed. A, B) Tilt angle between the BIg51 (green) and BIg52 (orange) domains in the crystal structure (A), and a tilted conformation observed during the MD simulations (B). The tilt angle (depicted as black line) was defined between the Cα-atoms of residues ^6^P and ^9^S of BIg51 and ^50^V of BIg52. C, D) Twist angle between the BIg51 (green) and BIg52 (orange) domains in the crystal structure (C), and a twisted conformation observed during the MD simulations (D). The twist angle (depicted as black line) was defined as torsion angle between the Cα-atoms of residues ^9^S and ^91^I of BIg51 and ^7^E and ^50^V of BIg52. E) Effect of mutation of the type I and II site on the geometry of the BIg51-52 domain pair. Structures were defined as tilted or twisted, if the respective interdomain angle deviates by more than 15 degree from the conformation present in the crystal structure. See A)-D) for the definition of the interdomain angles. All simulations were performed for BIg50-52. BIg50 has been omitted in the presentation for reasons of clarity. Ca^2+^ ions are indicated by spheres.

In order to assess the role of Ca^2+^ ions for the conformational stability, molecular dynamics (MD) simulations of WT and mutant SiiE were performed. We focused on BIg domains 50–52 for the following reasons: (i) a high-resolution crystal structure is available for this portion of SiiE, (ii) the fragment is sufficiently small to allow for extensive MD simulations, and (iii) the role of Ca^2+^-binding sites in BIg50-52 constructs was experimentally investigated (Fig 1). The role of Ca^2+^ ions was assessed from inspection of the tilt and twist angles defining the relative orientation of BIg 51 and 52 (Fig 4A–4D). The percentage of tilted or twisted structures detected over the simulation time is summarized in Fig 4E. A comparison of WT and mutant forms revealed that mutation of both type I and type II sites caused an enhanced tilting of the structure and thus a less extended domain arrangement compared to the WT structure. The stronger effect was observed for the type I site, which is consistent with the direct location in the domain interface. Notably, the concomitant mutation of both sites had an additive effect resulting in the highest portion of tilted structures among all systems investigated. Mutation of type I and type II site did not only affect tilting, but also resulted in an enhanced twisting of the domain pair (Fig 4E). However, in contrast to tilting, type I and type II site had a similar effect on domain twisting and there was no additive effect upon mutation of both sites. These data support the role of Ca^2+^-binding sites in increasing the rigidity of SiiE.

**Fig 5:**
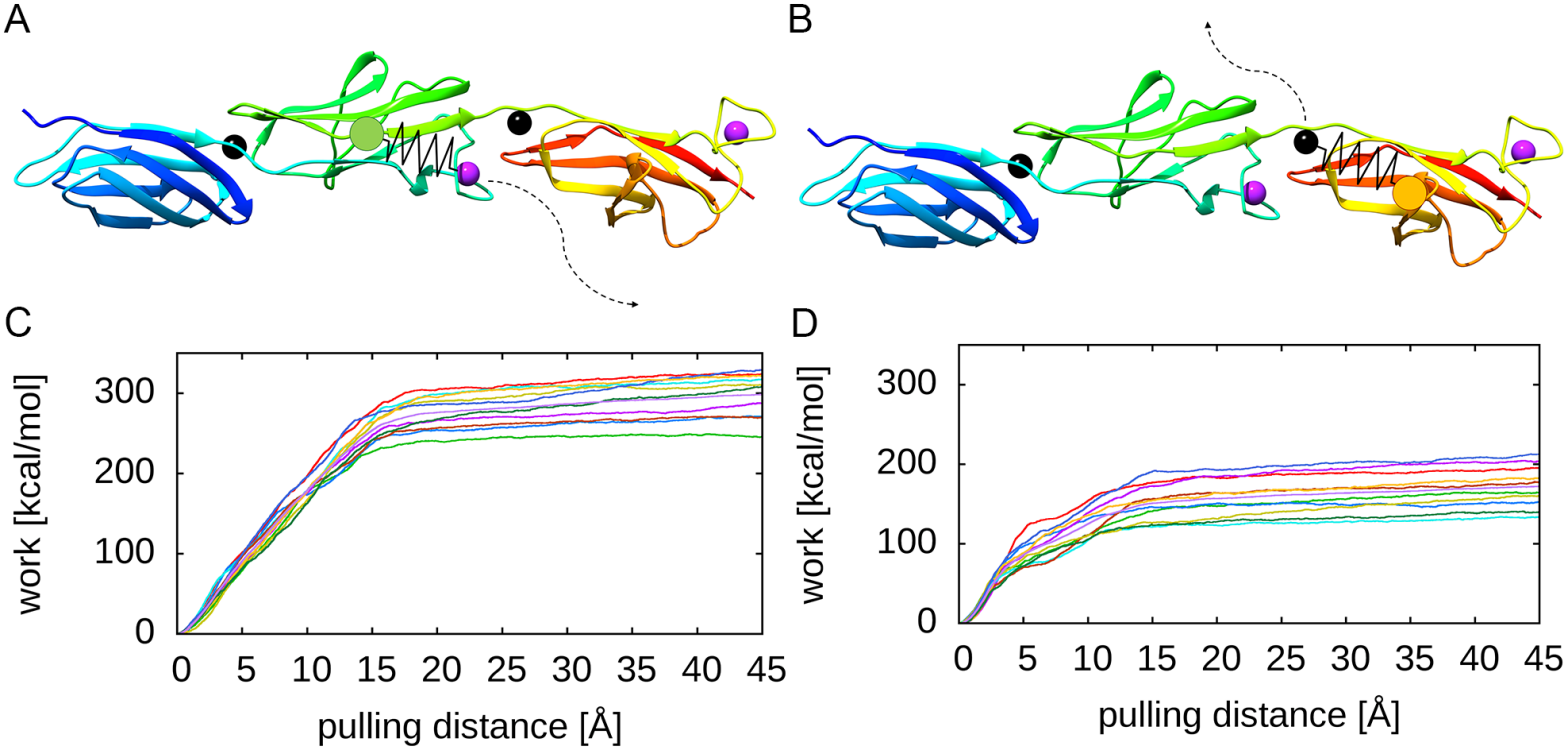
Steered molecular dynamics simulations for displacement of Ca^2+^ ions in SiiE. A) Setup for pulling the Ca^2+^ ion out of the type II site. A spring-like restraint (black zigzag line) was defined between the BIg51 center of mass (green circle) and the Ca^2+^ ion. The exit pathway of the ion from the binding site is schematically shown as dashed line. SiiE BIg domains 50, 51, and 52 are colored in blue, green, and orange, respectively. B) Setup for pulling the Ca^2+^ ion out of the type I site. In contrast to the setup in panel (A), the spring-like restraint was defined between the BIg52 center of mass (orange circle) and the Ca^2+^ ion. C, D) Work required to pull the Ca^2+^ ion out of the type II site (C) or the type I site (D). Results of 10 independent simulations are shown in different colors.

To assess the relative Ca^2+^ binding affinities of the type I and type II sites, steered molecular dynamics (SMD) simulations were performed. The setup is schematically depicted in Fig 5A and 5B. The Ca^2+^ ions were independently removed from both sites and 10 simulations were performed for each site. For the type II site, all 10 work plots display an overall similar shape (Fig 5C). Up to a distance of ~ 15 Å, the work linearly increases reflecting the disruption of the interactions between the Ca^2+^ ion and its protein ligands. For larger distances there is only a marginal further increase of the work, indicating that dissociation is almost complete at a distance of ~ 15 Å. The work required in the 10 SMD runs of the type II site ranges from 246–329 kcal x mol^-1^. For the type I sites, the qualitative appearance of the curves is similar (Fig 5D); however, less work is required for the removal of the ion from this site. The resulting work ranges from 134–212 kcal x mol^-1^, which is significantly lower than for the type II site.

### Role of Ca^2+^-binding sites in SiiE for conformation, stability and thermal-induced aggregation properties of SiiE

**Fig 6:**
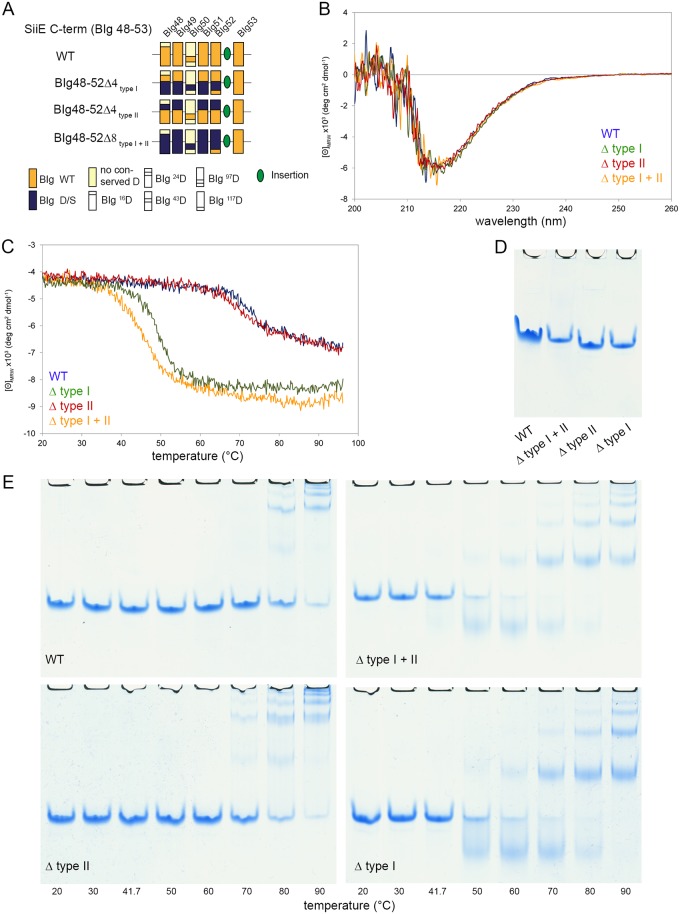
Role of Ca^2+^-binding sites in SiiE for folding, thermal stability and aggregation properties of SiiE. A) Schematic overview of the constructs containing the C-terminal region of SiiE which were used to examine the impact of D/S exchanges on the folding and stability of the protein *in vitro*. B) Circular dichroism (CD) spectra in the far UV region of SiiE_Cterm_ WT and mutant forms with mutation of type I, type II, or type I and type II Ca^2+^-binding sites. C) Thermal scanning CD analysis of the stability of the SiiE_Cterm_ variants. D) Electrophoretic motility on native PAGE of SiiE_Cterm_ WT and Ca^2+^-binding site mutants. E) The aggregation behavior of SiiE_Cterm_ WT and variant forms was investigated by incubating samples of each protein at increasing temperatures and analysis on native PAGE.

SiiE variants with altered type I and type II Ca^2+^-binding sites in the C-terminal moiety of SiiE (SiiE_Cterm_) were characterized in detail in *in vitro* experiments. These variants encompass BIg domains 48–53, the insertion and the C-terminal segment that includes the secretion signal (Fig 6A). We have previously shown that recombinant protein production of a fragment that covers BIg domains 50 to 52 in *E*. *coli* and subsequent purification of the fragment without the addition of any Ca^2+^ ions leads to a protein sample in which all Ca^2+^-binding sites are fully occupied [8]. This observation was corroborated not only by the final electron density map of the solved crystal structure, but also by analyzing in detail the anomalous scattering signal and *via* X-ray fluorescence measurements [8]. Here, we now used inductively coupled plasma—atom emission spectroscopy (ICP-AES) to verify the presence and/or absence of Ca^2+^ ions in protein variants that were produced following a similar purification protocol as previously described for the BIg domains 50 to 52 protein fragment [8]. In case of WT SiiE_Cterm_ the occurrence of 8 to 10 Ca^2+^-binding sites was expected. In the ICP-AES experiment the protein was analysed at a concentration of 6.5 mg x ml^-1^ (88.1 μM), which results in a theoretical maximum calcium content of 28–35 μg x ml^-1^ (705.2–881.5 μM). The experimentally determined calcium concentration was 23 μg x ml^-1^ which amounts to 81.8% to 65.44% of the expected theoretically maximum content (Table 1, S4 Fig). Variant SiiE_Cterm_ with type I and type II Ca^2+^-binding sites mutated (SiiE_Cterm_ BIg48-52Δ8_type I + II_) was measured at a concentration of 4.2 mg x ml^-1^ (57.63 μM). For this variant, a calcium signal below the 0.5 μg x ml^-1^ calibration standard and outside the calibration range was detected (S4 Fig). Thus, no calcium binding is observed for variant SiiE_Cterm_ BIg48-52Δ8_type I + II_ (Table 1). These measurements show that in case of the WT protein the Ca^2+^-binding sites are almost fully occupied in the recombinantly produced protein sample whereas the substitution of defined aspartic residues against serines in the SiiE_Cterm_ BIg48-52Δ8_type I + II_ results in a protein that is devoid of any calcium binding. Conversely, variants with some of the Ca^2+^-binding sites disrupted should display reduced Ca^2+^ binding if one assumes the absence of any cooperativity between the binding sites.

**Table 1:** Analysis of calcium content of SiiE_Cterm_ WT and BIg48-52Δ8_type I + II_ by ICP-AES.

	WT	SiiE_Cterm_ BIg48-52Δ8_type I + II_
Conc1^1^	23.01 μg x ml^-1^	-0.34 μg x ml^-1^
Conc2^1^	21.14 μg x ml^-1^	-0.41 μg x ml^-1^
Conc3^1^	25.21 μg x ml^-1^	-0.47 μg x ml^-1^
Conc Mean^2^	23.12 μg x ml^-1^	-0.41 μg x ml^-1^
Conc SD^3^	2.04 μg x ml^-1^	0.06 μg x ml^-1^
Conc RSD^4^	8.8%	15.7%

^1^ Concentration determined by individual measurements

^2^ Concentration mean

^3^ Standard deviation of mean

^4^ Relative standard deviation

To experimentally address individual roles of type I and type II Ca^2+^-binding sites for the conformational stability of SiiE, we performed circular dichroism (CD) measurements. Highly similar FarUV spectra were recorded for all variants, namely the SiiE_Cterm_ construct with WT sequence, mutations of type I, type II and of both type I and type II Ca^2+^-binding sites (Fig 6B). Estimation of the secondary structure content using the BeStSel server suggests that the SiiE_Cterm_ wild-type variant consist of around 50% β-sheets, 10% turns and 40% others (e.g. random coil). These values are close to those derived from the available SiiE BIg-domain crystal structure (BIg domains 50–52, PDB entry: 2YN5). This suggests that the so far structurally uncharacterized domains (BIg domains 48–49 and BIg domain 53) display similar secondary structures as domains 50 to 52. Most importantly, however, the highly similar spectra and concomitant results from the secondary structure analysis of the variants studied here demonstrate that the secondary structure composition of the proteins is not altered, thus excluding pronounced mis- or unfolding, when mutating the type I and/or type II Ca^2+^-binding sites (S4 Table). The thermal scanning CD measurements revealed distinct effects of type I and type II site mutations on the conformational stability of SiiE_Cterm_ (Fig 6C). All protein variants exhibit a decrease in ellipticity upon heating, which suggests that instead of a thermally induced unfolding an increased secondary structure and/or β-sheet formation via aggregation occurs. However, the magnitude of this transition is lower for SiiE_Cterm_ WT and SiiE_Cterm_ BIg48-52Δ4_type II_ than for SiiE_Cterm_ BIg48-52Δ4_type I_ and SiiE_Cterm_ BIg48-52Δ8_type I + II_. Also, T_onset_ of these structural changes is higher for WT and Δ4_type II_ variants at 72°C and 69°C, respectively, than for Δ4_type I_ and Δ8_type I + II_ variants at 50°C and 45°C, respectively. Thus, while the thermal scanning CD curve of SiiE_Cterm_ BIg48-52Δ4_type II_ resembles that of SiiE_Cterm_ WT, the spectra of proteins with mutations of type I and both type I and II sites indicated that the proteins are more prone to aggregation.

To further investigate the conformational stability, the SiiE_Cterm_ variants were subjected to native PAGE (Fig 6D and 6E). All variants covering the C-terminal moiety of SiiE migrated as a single band under mild conditions (Fig 6D). To further investigate the aggregation behavior, individual samples of each variant were incubated at increasing temperatures and subsequently analyzed by native PAGE. SiiE_Cterm_ WT was resistant to aggregation up to 80°C and SiiE_Cterm_ BIg48-52Δ4_type II_ behaved similar to WT protein, although pronounced aggregation was detected at a slightly lower temperature (Fig 6E). A ladder-like pattern indicates that SiiE_Cterm_ BIg48-52Δ8_type I + II_ started to form oligomers and aggregated already at 50°C. Aggregation of SiiE_Cterm_ BIg48-52Δ4_type I_ resembled SiiE_Cterm_ BIg48-52Δ8_type I + II_, although slightly delayed (Fig 6D). The analysis of the aggregation behavior therefore reflects the results of the thermal scanning CD measurements. Faster migrating protein species are visible in the native PAGE of the Δ4_type I_ and Δ8_type I + II_ mutants. To control whether unwanted proteolysis might have caused the occurrence of these so-called lower bands, SDS-PAGE analysis was performed for SiiE_Cterm_ samples after incubation at various temperatures (S5 Fig). Neither WT SiiE_Cterm_ nor any of the mutant forms indicate a temperature-dependent occurrence of proteolytic fragments. The increased migration behavior thus results from a partial collapse of the expected linear overall structure of SiiE into a more globular domain arrangement as the result of the removal of the type I Ca^2+^-binding sites that are located in the interface between BIg domains.

Next, the conformation and compactness of the SiiE_Cterm_ variants was probed by limited proteolysis using α-Chymotrypsin and Proteinase K (S6 Fig). Multi-domain proteins with flexible domain surface loops and/or interdomain linkers are expected to be more prone to proteolytic cleavage than proteins with very rigid domain architecture. Resistance against proteolytic cleavage by α-Chymotrypsin was clearly reduced for SiiE_Cterm_ BIg48-52Δ8_type I + II_ in comparison to WT protein or protein with only type I or type II binding site mutations. This suggests that both types of Ca^2+^-binding sites help to stabilize the fold against proteolytic degradation. The effect is possibly enhanced by the close spatial proximity of the two binding sites [8]. Resistance against proteolytic cleavage by Proteinase K was also reduced most for SiiE_Cterm_ BIg48-52Δ8_type I + II_ similarly to the proteolysis using α-Chymotrypsin. However, the stability of the individual type I or type II Ca^2+^-binding site mutants was also decreased, although to a lesser extent than for the variant with both binding sites mutated.

### Type I Ca^2+^-binding sites are more important for secretion than type II Ca^2+^-binding sites

**Fig 7:**
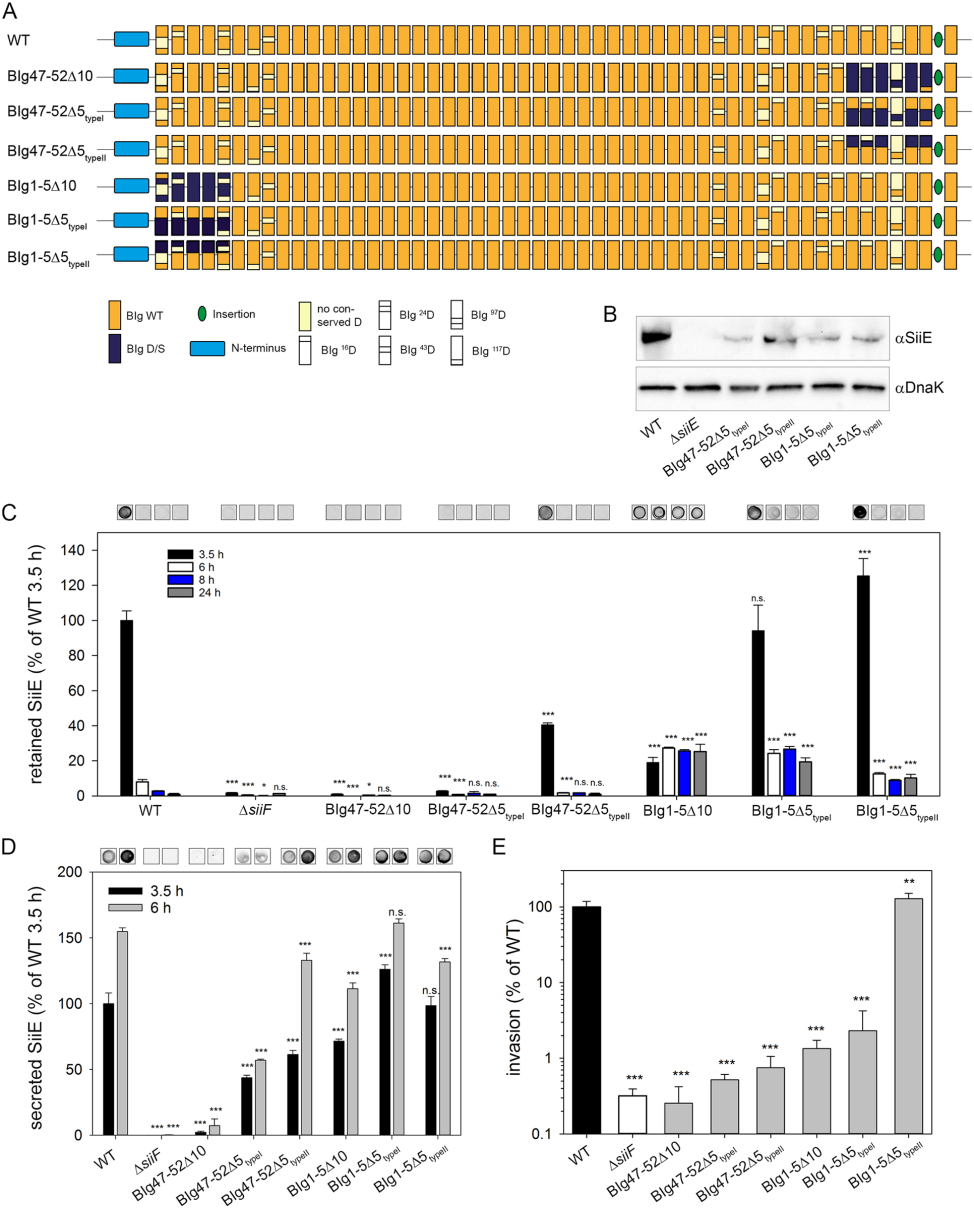
Functional dissection of type I and type II Ca^2+^-binding sites. SiiE was mutated in 5 consecutive BIg domains in the N-terminal (BIg1-5) or C-terminal (BIg47-52) region in 10 Ca^2+^-binding sites (D10), 5 type I Ca^2+^-binding sites only (Δ5_typeI_), or 5 type II Ca^2+^-binding sites only (Δ5_typeII_). A) Schematic overview of D/S exchanges introduced for deletion of Ca^2+^-binding sites in BIg1-5 or BIg 47–52. B) Western blot for analyses of synthesis of mutant forms of SiiE. C) Amounts of SiiE retained on the bacterial surface at 3.5 h, 6 h, 8 h and 24 h of subculture. D) Amounts of SiiE secreted in culture supernatant after 3.5 h and 6 h of subculture. E) SiiE-dependent invasion of polarized epithelial MDCK cells. Analyses of synthesis, surface retention and secretion and SiiE-dependent invasion of polarized cells were performed as described for Fig 2.

Finally, we set out to functionally dissect the roles of type I and type II Ca^2+^-binding sites in SiiE. We generated mutant alleles by site-directed mutagenesis for single aa exchanges in BIg51 and BIg52 (S7 Fig), or exchanges of all residues of either the type I or the type II Ca^2+^-binding sites within BIg52, within BIg47-52, or BIg1-5 (Fig 7).

Mutation of single aspartate residues or D/S exchanges in single Ca^2+^-binding sites in chromosomal *siiE* did not affect SiiE synthesis, surface retention, secretion, or SiiE-dependent invasion (S7B, S7C, S7D and S7E Fig). In contrast, if either type I or type II Ca^2+^-binding sites are missing within the five C-terminal BIg domains 47–52, invasion is reduced to the level of the negative control (Fig 7E). For both mutants, retention (Fig 7C) and secretion (Fig 7D) was reduced. This reduction was more pronounced for SiiE BIg47-52Δ5_type I_ than for SiiE BIg47-52Δ5_type II_. Retention was fully abolished for SiiE BIg47-52Δ5_type I_, as well as for BIg47-52Δ10 for all time points tested, while secretion was reduced to 50% of WT SiiE. For SiiE BIg47-52Δ5_type II_ retention after 3.5 h of subculture was reduced to 40% of WT SiiE and after 6 h and later time points no surface retention was detected, similar to WT SiiE.

Removal of type I or type II Ca^2+^-binding sites in BIg1-5 had only minor effects on retention and secretion of SiiE. In contrast to SiiE BIg1-5Δ5_type II_, SiiE BIg1-5Δ5_type I_ was retained at late time points at a level similar to BIg1-5Δ10 (8 and 24 h). SiiE BIg1-5Δ5_type II_ was also retained at late time points, but to a lesser extent than SiiE BIg1-5Δ5_type I_ or SiiE BIg1-5Δ10. The mutation of five type I sites in BIg1-5 resulted in highly (43.5-fold) reduced invasion, while removal of five type II Ca^2+^-binding sites in the same moiety (BIg1-5Δ5_type II)_ did not reduce invasion of polarized epithelial cells (Fig 7E). These results suggest distinct roles of type II Ca^2+^-binding sites in N- and C-terminal portions of SiiE.

**Fig 8:**
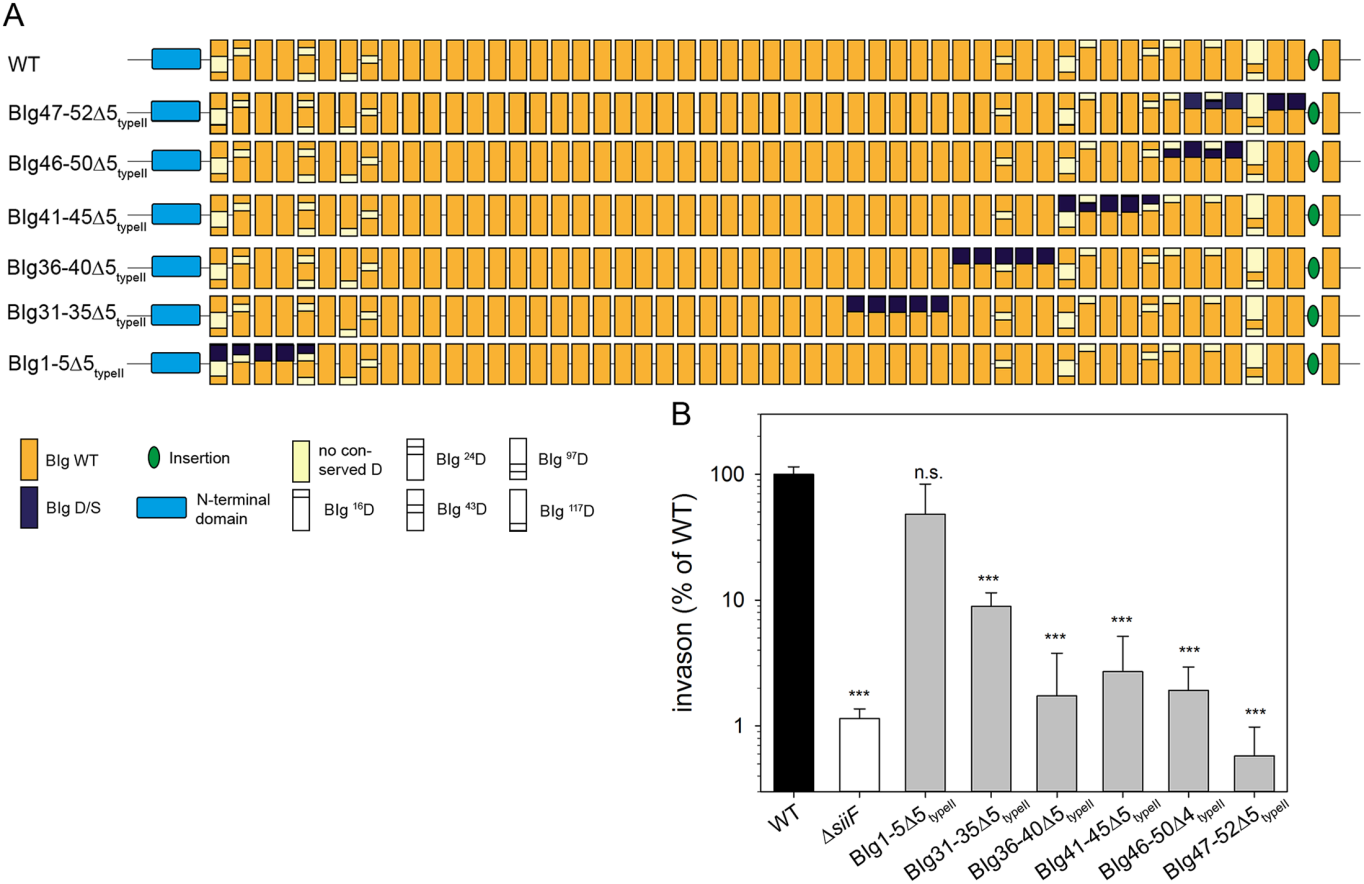
The position of type II Ca^2+^-binding sites is critical for SiiE function. The type II Ca^2+^-binding sites of 5 consecutive BIg domains (Δ5_typeII_) were removed by D/S exchanges in various positions of SiiE as indicated (A). B) SiiE-dependent invasion of polarized epithelial MDCK cells by *Salmonella* strains expressing WT and mutant alleles of SiiE. Analysis of SiiE-dependent invasion of polarized cells was performed as described for Fig 2.

To further analyze the role of type II Ca^2+^-binding sites for function of SiiE, we removed type II Ca^2+^-binding sites by D/S exchanges in BIg31-35, BIg 36–40, BIg 41–45, or BIg 46–50. The invasion of MDCK cells of strains expressing these mutant forms of *siiE* was compared to invasion by strains with WT SiiE, SiiE BIg1-5Δ5_type II_ and SiiE BIg47-52Δ5_type II_ (Fig 8). We observed that strains producing SiiE with type II sites removed in BIg31-35, BIg 36–40, BIg 41–45, BIg 46–50 or BIg47-52 all exhibited reduced invasion compared to strains with SiiE WT or SiiE BIg1-5Δ5_type II_. The reduction of invasion was pronounced if BIg domains in the C-terminal region were affected, and smallest reduction of invasion was observed for the strain with SiiE BIg30-35Δ5_type II_.

The fact that SiiE BIg1-5Δ5_type II_, but not SiiE BIg47-52Δ5_type II_ still mediates binding to, and invasion of MDCK cells indicates that type II Ca^2+^-binding sites are more important for the correct local conformation of the protein, which might be necessary for proper binding. We conclude that in SiiE BIg1-5Δ5_type II_ the C-terminal part is correctly folded and can mediate binding to the host cell, while this is not the case for SiiE BIg47-52Δ5_type II_.

## Discussion

Our comprehensive mutational and functional analyses revealed a role of conserved aspartate residues in BIg domains of SiiE in secretion and adhesin function of this giant adhesin. An increasing number of exchanges of conserved aspartate residues resulted in decreased secretion of SiiE. This observation was made for a C-terminal plasmid-encoded portion of SiiE, as well as for chromosomally encoded variants of SiiE with the same amino acid exchanges. For chromosomally encoded SiiE BIg52Δ2, the secretion was reduced. This reduced amount of secreted SiiE was not associated with higher levels of SiiE retention, or reduced SiiE-dependent invasion. Also, 5 D/S exchanges in BIg2Δ2 or BIg40Δ2 in chromosomally encoded SiiE did not lead to reduced invasion. Based on these results we conclude that the number of functional SiiE molecules on the bacterial surface is still high enough to mediate apical adhesion and subsequent invasion.

**Fig 9:**
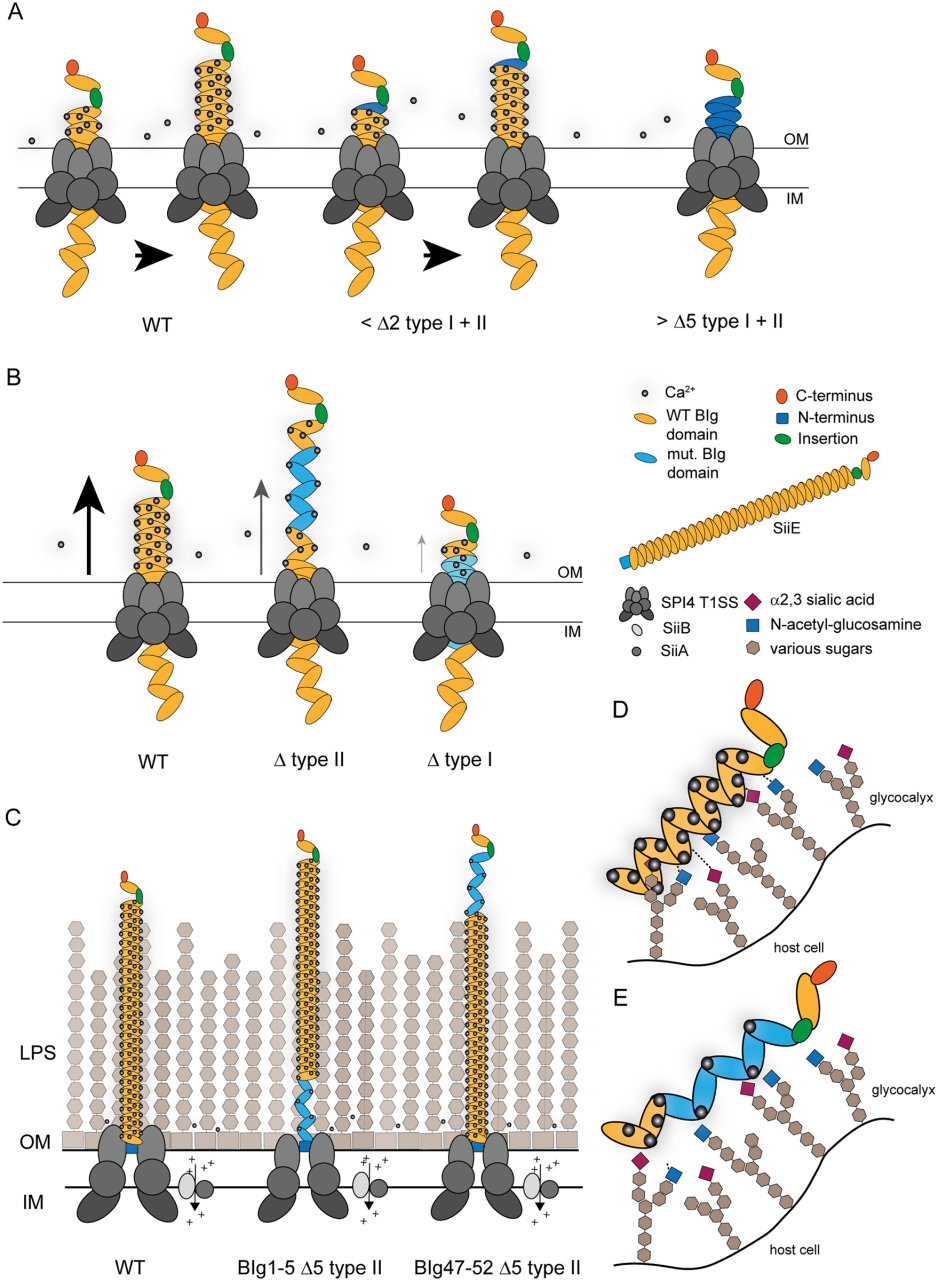
Models for the roles of Ca^2+^-binding sites in giant adhesin SiiE. A) The binding of Ca^2+^ ions to BIg domains supports secretion by the T1SS. If only a few Ca^2+^-binding sites are absent, the defect is compensated by the remaining Ca^2+^-binding sites in BIg domains preceding or following the mutated region. Removal of Ca^2+^-binding sites in several consecutive BIg domains results in block of secretion. B) Role of type I and type II Ca^2+^-binding sites. Type II sites support proper conformation, while type I sites support secretion and rigidity. If type I sites are missing, secretion ceases, SiiE does not protrude the O-antigen layer, thus is unable to mediate adhesion to polarized cells. C) Role of type II Ca^2+^-binding sites in various positions of SiiE. BIg1-5 Δ type II has a functional C-terminal part, which protrudes beyond the O-antigen layer and can bind to target structures on host cells. Mutations BIg47-52 Δ type II render the C-terminal moiety of SiiE non-functional in mediating binding and subsequent invasion. D), E) Model for the effect of type II Ca^2+^-binding sites in interaction of SiiE with cognate glycostructures. The binding of Ca^2+^ ions leads to a conformation of BIg domains that enables the interaction with N-acetyl-glucosamine and/or α2,3-linked sialic acid. Multiple BIg domains may contribute to the binding, resulting in a sum of weak interactions between SiiE and glycostructures on the host cell apical membrane. The interactions are ablated upon removal of Ca^2+^ binding in BIg domains.

We found that deletion of two Ca^2+^-binding sites by 5 D/S exchanges in the N- or C-terminal parts of SiiE showed no or only mild phenotypic difference, indicating that the remaining Ca^2+^-binding sites in adjacent domains can compensate for a certain degree of loss of Ca^2+^-binding properties of SiiE. Upon deletion of 5 or more Ca^2+^-binding sites, we observed loss of SiiE retention, dramatically decreased amounts of secreted SiiE and attenuated invasion. Thus, the lack of 5 Ca^2+^-binding sites in the C-terminal portion of SiiE could not be compensated by the function of residual domains. We propose a model in which binding of extracellular Ca^2+^ ions promotes directionality in the secretion of SiiE (Fig 9A). If many consecutive D residues are missing, Ca^2+^ binding is ablated and the secretion is reduced. Lack of a few Ca^2+^-binding sites is not critical since Ca^2+^ ions will bind to the next available Ca^2+^-binding site of BIg domains that are already outside of the T1SS. If too many Ca^2+^-binding sites are missing, the next available Ca^2+^-binding sites are still within the channel of the T1SS and not accessible for the extracellular Ca^2+^ ions (Fig 9A). Dependent on the position of the missing Ca^2+^-binding sites within SiiE, secretion of SiiE is arrested at a certain stage. If Ca^2+^-binding sites were removed in BIg1-5, SiiE was also surface-retained at 6 h of subculture and later, indicating that the secretion process stopped. Exchanges in the C-terminal moiety, namely BIg47-52Δ10 led to secretion stalling early in the process of secretion, so that surface expressed or secreted SiiE was highly reduced.

If fusion proteins covering a short portion of SiiE are investigated (Fig 1), lack of already a few Ca^2+^-binding sites leads to a dramatic decrease in secreted SiiE. Possibly, not only the number of lacking Ca^2+^-binding sites is responsible for this, but also the overall number of available Ca^2+^-binding sites.

Thomas *et al*. [13] recently described a Ca^2+^ driven folding that may facilitate secretion in *E*. *coli* pro-HlyA. HlyA contains RTX motifs that bind Ca^2+^. If no Ca^2+^ is bound, or if Ca^2+^ is chelated by e.g. EDTA, the protein remains unfolded [13, 21, 22]. A similar mechanism could be considered for secretion of SiiE, with binding of extracellular Ca^2+^ ions initially facilitating secretion and later supporting the proper conformation and interaction with ligands. Such an interaction could, for example, occur with the carboxyl group of SiiE ligand α2,3-linked sialic acid.

From other T1SS substrates it is known that they are secreted in an unfolded state. For SiiE, the intracellular folding rate is not the most critical parameter for the initialization of the secretion process, as seen for the *E*. *coli* HlyA system [13] (S3 Fig). Since the SPI4-encoded T1SS possesses two unique accessory proteins SiiA and SiiB, these proteins could promote the start of SiiE release until the protein becomes accessible to extracellular Ca^2+^ ions. SiiE has to be transiently retained on the bacterial surface in order to function as an adhesin. The proton channel formed by SiiA and SiiB or possibly other interactions between subunits of the T1SS could act as the retention signal, which has to be stronger than the extracellular Ca^2+^ ions. More experiments are needed to fully understand the retention process of this exceptional adhesin.

Why does SiiE possess two distinct types of Ca^2+^-binding sites? Conserved D residues forming type I Ca^2+^-binding sites can be found in other bacterial adhesins or secreted enzymes, like BapA from *Salmonella enterica*, LapF from *Pseudomonas putida*, or the PKD domain from the plant cell wall-targeting endoglucanase of *Clostridium thermocellum* [8, 23–25]. In contrast, type II Ca^2+^-binding sites are specific for SiiE. Since type I and type II Ca^2+^-binding sites are structurally different, also distinct functions have to be considered. We speculate that type II Ca^2+^-binding sites are required for proper fine-tuning of the conformation of SiiE, while type I Ca^2+^-binding sites promote an overall rigidification of the tertiary structure of SiiE and thereby secretion through the T1SS. To elucidate the impact of type I and type II Ca^2+^-binding sites, several binding sites within the C-terminal portion were exchanged. All recombinantly produced C-terminal variants showed similar CD spectra showing that the secondary structure content was not altered in these variants. However, compared to type II sites, removal of type I sites had more dramatic effects on secretion and retention, independent of the region in SiiE in which the mutations were introduced. This observation would be in line with a role of type I sites in supporting secretion (Fig 9B). Interestingly, mutations BIg47-52Δ5_type I_, and BIg1-5Δ5_type I_ led to loss of function of SiiE in supporting invasion of polarized cells. Loss of SiiE function was observed for mutation BIg47-52Δ5_type II_, while mutation BIg1-5Δ5_type II_ only caused minute alteration in invasion of MDCK cells. Thus, type II Ca^2+^-binding sites in the N-terminal region of SiiE are dispensable for secretion and surface expression of SiiE.

BIg domains of LapF and BapA are more similar to SiiE BIg50, harboring only a type I Ca^2+^-binding site [8]. Until now, no distinct role for BIg50 could be identified. Since it is much shorter than the other SiiE BIg domains, one Ca^2+^-binding site could be sufficient to stabilize this domain, whereas longer BIg domains need two Ca^2+^-binding sites to be stabilized.

We previously reported that Ca^2+^ ions stabilize a rigid, linear conformation of SiiE, suggesting a role of Ca^2+^-binding sites for the overall protein stability [8]. The MD simulations of the present study showed that mutations of type I as well as type II sites destabilize SiiE and cause more frequent deviations from the geometry of the crystal structure. In particular, changes of the tilt angle result in less extended conformations (Fig 4B) that are expected to exhibit a reduced SiiE functionality. It is also interesting to note that the majority of the tilt angles observed during simulation are in the range of 15° to 30°. These rather small tilt angles suggest that larger deviations from the extended geometry require kinking at multiple sites at the same time. This is in line with the experimental data that mutations of multiple type I sites are required in order to disturb SiiE function. The observation that tilting is predominantly enhanced by mutation of the type I site suggests that these sites might also be more critical for the structural rigidification of SiiE and is in line with the observation that disruption of the type I sites leads to an earlier onset of temperature-induced aggregation of SiiE. Although the work calculated by SMD simulations cannot readily be converted into binding affinities, it suggests that type II sites exhibit higher Ca^2+^ affinity compared to type I sites (Fig 5). This is in line with a sequential mechanism of Ca^2+^ binding during secretion, in which initially the type II sites get occupied (probably during the folding of the individual domains), and subsequently Ca^2+^ binding of the type I sites stabilizes the extended domain arrangement of SiiE.

We propose that local conformational distortions in the C-terminal BIg domains due to the BIg47-52Δ5_type II_ mutations may result in loss of binding to host cell ligands, despite sufficient amounts of SiiE being surface expressed. For SiiE BIg1-5Δ5_type II_, the C-terminally located BIg domains are correctly folded and proficient in ligand binding. This model also implies that BIg domains located in the N-terminal and perhaps also in the central portion of SiiE are not directly involved in binding to ligands on apical membranes of host cells (Fig 9C, 9D and 9E). Although Ca^2+^ ions promote SiiE secretion and folding, we do not assume that they are also directly involved into binding to the target structure. SiiE binds to GlcNAc- and sialic acid-containing structures [5]. Griessl *et al*. [8] also showed that the majority of SiiE BIg domains possess a conserved tryptophan residue, however, exchanges of this aromatic residue in BIg50-52 did not influence SiiE specific phenotypes (S2 Fig). Future mutational and functional analyses may identify the residues in C-terminal BIg domains of SiiE that directly contribute to the interaction with ligands.

The 'C-terminus first secretion' of T1SS substrate proteins has been formally demonstrated for HlyA [26]. The C-terminal RTX domain of *B*. *pertussis* CyaA has 5 repeat blocks containing GGxGxDxxx motifs that form β-rolls coordinating Ca^2+^ binding. A total of 40 Ca^2+^ ions bound per CyaA molecule have been estimated [27]. Recent analyses of T1SS secretion of CyaA and related RTX toxins demonstrated a vectorial push-ratchet mechanism [12, 14]. In the bacterial cytosol, the RTX domain is intrinsically disordered. After exit of the T1SS duct, the β-rolls sequentially bind Ca^2+^ at the outside of the cell envelope, initiate folding of the C-terminal domain and thereby provide directionality and secretion support for the rest of the protein [28, 29]. The proposed model is in line with earlier findings, namely that ATP-hydrolysis and membrane potential are only required for the initiation of secretion, while further secretion is driven by the folding of portions of the substrate protein that have left the T1SS [11, 14]. Interestingly, a recent analysis revealed that HlyA secretion efficiency is independent from Ca^2+^ concentration ranging from 0 to 5 mM Ca^2+^ in the external medium [30]. This observation would argue against a role of Ca^2+^ in supporting T1SS secretion of HlyA, however, the local Ca^2+^ pool in the outer membrane of secreting bacteria also has to be considered.

Although Ca^2+^ binding by type I and type II Ca^2+^-binding sites in SiiE is mediated by structurally distinct motifs, we propose a function similar to RTX repeats regarding disordered-to-folded transition and directionality of secretion. While Ca^2+^-binding sites in CyaA are restricted to the C-terminal RTX domain, Ca^2+^-binding sites are present in all BIg domains of SiiE. This would be in line with a dual function of Ca^2+^ binding in promoting secretion, as well as stabilizing the ligand-binding competent overall conformation of SiiE.

## Materials and methods

### Bacterial strains and culture conditions

*Salmonella enterica* serovar Typhimurium (*S*. Typhimurium) NCTC 12023 was used as wild-type strain in this study and all mutant strains are isogenic to this strain. The characteristics of strains used in this study are listed in Table 2. Bacterial strains were routinely grown in LB broth or on LB agar containing antibiotics if required for selection of specific markers. The Ca^2+^ concentration in LB media is not defined. Carbenicillin or kanamycin were used at 50 μg x ml^-1^, and tetracycline or chloramphenicol were added to a final concentration of 20 or 10 μg x ml^-1^ respectively, if required for the selection of phenotypes or maintenance of plasmids.

**Table 2:** Bacterial strains used in this study.

Designation	genotype	relevant characteristics	reference
NCTC 12023	wild type		lab stock
MvP599	Δ*siiE*::FRT		[31]
MvP812	Δ*siiF*::FRT		[31]
MvP1986	*siiE*_825-1287_::I-*SceI aph*		this study
MvP1989	*siiE*_12177-12624_::I-*SceI aph*		this study
MvP1970	*siiE* BIg47-52::I-*Sce*I *aph*		this study
MvP2105	*siiE*_708-2173_::I-*SceI aph*		this study
MvP2110	*siiE*_15232-16080_::I-*SceI aph*		this study
MvP2028	*siiE*_15475-16080_::I-*SceI aph*		this study
MvP2114	*siiE* BIg50_W74F_	1 tryptophan mutated	this study
MvP2115	*siiE* BIg51_W74F_	1 tryptophan mutated	this study
MvP2123	*siiE* BIg52_W74F_	1 tryptophan mutated	this study
MvP2126	*siiE* BIg51/52_W74F_	2 tryptophan mutated	this study
MvP2116	*siiE* BIg50/51_W74F_	2 tryptophan mutated	this study
MvP2124	*siiE* BIg50/52_W74F_	2 tryptophan mutated	this study
MvP2125	*siiE* BIg50-52_W74F_	3 tryptophan mutated	this study
MvP2031	*siiE* BIg51_D117S_	no Ca^2+^-binding sites mutated	this study
MvP2032	*siiE* BIg52_D16S_	no Ca^2+^-binding sites mutated	this study
MvP2033	*siiE* BIg52_D24S_	no Ca^2+^-binding sites mutated	this study
MvP2034	*siiE* BIg52_D43S_	no Ca^2+^-binding sites mutated	this study
MvP2035	*siiE* BIg52_D97S_	no Ca^2+^-binding sites mutated	this study
MvP2117	siiE BIg47-52_WT restored_	no Ca^2+^-binding sites mutated	this study
MvP2037	*siiE* BIg51_D117S_ BIg52_D43S D97S_	Δ1 type I Ca^2+^-binding site mutated	this study
MvP2036	*siiE* BIg52_D16S D24S_	Δ1 type II Ca^2+^-binding sites mutated	this study
MvP2001	*siiE* BIg1_D117S_ BIg2_D16S D24S D43S D96S_	Δ2 type I + II Ca^2+^-binding sites mutated	this study
MvP2002	*siiE* BIg39_D117S_ BIg40_D16S D24S D43S D96S_	Δ2 type I + II Ca^2+^-binding sites mutated	this study
MvP1881	*siiE* BIg52 Δ2	Δ2 type I + II Ca^2+^-binding sites mutated	this study
MvP1983	*siiE* BIg47 Δ1 BIg51-52 Δ4	Δ5 type I + II Ca^2+^-binding sites mutated	this study
MvP1985	*siiE* BIg47 Δ2 BIg51-52 Δ4	Δ6 type I + II Ca^2+^-binding sites mutated	this study
MvP2149	*siiE* BIg1-5 Δ10	Δ10 type I + II Ca^2+^-binding sites mutated	this study
MvP1984	*siiE* BIg47-52 Δ10	Δ10 type I + II Ca^2+^-binding sites mutated	this study
MvP2294	*siiE* BIg1-5 Δ5 type I	Δ5 type I Ca^2+^-binding sites mutated	this study
MvP2168	*siiE* BIg47-52 Δ5 type I	Δ5 type I Ca^2+^-binding sites mutated	this study
MvP2295	*siiE* BIg1-5 Δ5 type II	Δ5 type II Ca^2+^-binding sites mutated	this study
MvP2469	*siiE* BIg31-35 Δ5 type II	Δ5 type II Ca^2+^-binding sites mutated	this study
MvP2470	*siiE* BIg36-40 Δ5 type II	Δ5 type II Ca^2+^-binding sites mutated	this study
MvP2471	*siiE* BIg41-45 Δ5 type II	Δ5 type II Ca^2+^-binding sites mutated	this study
MvP2472	*siiE* BIg46-50 Δ5 type II	Δ5 type II Ca^2+^-binding sites mutated	this study
MvP2029	*siiE* BIg47-52 Δ5 type II	Δ5 type II Ca^2+^-binding sites mutated	this study

### Cell culture

Madin-Darby Canine Kidney Epithelial (MDCK) cells are an immortalized cell line initially derived from renal tube of a cocker spaniel. MDCK Pf subclone used for the generation of polarized epithelial cell monolayers was kindly provided by Department of Nephroplogy, FAU Erlangen-Nürnberg. MDCK cells were used for the generation of polarized epithelial cell layer. Cell culture conditions were previously described by Wagner et al. [6]. Briefly, cells were cultures in MEM with Earle’s salts, 4 mM Glutamax, non-essential amino acids and 10% heat-inactivated fetal calf serum. The Ca^2+^ concentration in this medium is 1.8 mM.

### Cloning

The synthetic DNA fragments (GeneArt or IDT) were subcloned in blunt end restriction sites of the pJET1.2 vector backbone. The synthetic DNA fragment SiiE-BIg49_half_-52_D-S_ was first cloned into pWRG454 (pWSK29::P_siiA_GlucM43LM110L::siiE-BIg50-53) via *Hin*dIII, *Nhe*I digest and ligation. To obtain the whole SiiE-BIg49-52_D-S_, the second synthetic DNA fragment SiiE-BIg47-49_half D-S_ was cloned into pWSK29::P_siiA_GlucM43LM110L::siiE-BIg49_half_-52_D-S_ via *Hin*dIII digestion and ligation. Orientation was checked by *Pst*I, *Cla*I diagnostic digest and subsequent sequencing confirmed the construct.

### Site-directed mutagenesis

The Q5 site-directed mutagenesis kit (NEB) was used to create plasmid with exchanges in codons for single conserved aspartate residues or exchanges for type I or type II Ca^2+^-binding sites in SiiE. Primers included new recognition sites for restriction enzymes through silent mutations. After performing colony PCR, the PCR fragment was digested with an appropriate restriction enzyme to confirm the silent mutations. Clones have also been confirmed by sequencing.

### Construction of chromosomal exchanges of conserved aspartate residues

The construction of scar-less in-frame deletions in *siiE* using the I-*Sce*I site was previously described by Blank *et al*. [32] and the protocol modified by Hoffmann *et al*. [33] was applied here. Briefly, the I-*Sce*I *aph* resistance cassette was amplified from pWRG717 using primers with 20 bp homology and 40 bp 5’ overlap. The resistance cassette was inserted by Red-mediated recombination within the desired region. The plasmid pWRG730 was used instead of pKD46 and features a heat-inducible promoter for *red* genes. For preparing competent cells of WT [pWRG730], cells were grown in LB Cm^10^ to OD_600_ of 0.4–0.5 in a baffled flask in a shaking water bath at 150 rpm at 30°C. Subsequently, cells were immediately transferred to another water bath pre-heated to 42°C and incubated for 12.5 min at 100 rpm. After incubation of heat-induced cells on ice for 15 min, competent cells were prepared as described before [34]. After *Dpn*I-digestion and purification, the PCR product was electroporated into *Salmonella* and transformants were selected on LB Km^25^ agar plates. Transformants were checked by colony PCR and confirmed clones were streaked on LB Cm^10^ plates with or without 100 ng x ml^-1^ anhydrotetracycline (AHT, Sigma-Aldrich). Clones with the highest inhibition on AHT containing plates at 30°C were selected for further procedures.

PCR fragments were amplified from plasmids containing exchanges of conserved aspartate codons in *siiE* or synthetic DNA were used. These fragments contain 5’ overlaps which are homolog to the insertion site of the chromosomally integrated I-*Sce*I *aph* cassette. The strain containing the I-*Sce*I *aph* cassette and harboring pWRG730 was then transformed by electroporation with either purified PCR product or synthetic DNA fragments. Serial dilutions from 10^−1^ to 10^−4^ were plated on LB Cm^10^ plates containing 100 ng x ml^-1^ AHT and incubated overnight at 30°C. The next day, large colonies were re-streaked on LB Cm^10^ AHT^100^ plates. Clones were verified by testing sensitivity to kanamycin and by colony PCR.

### Gaussia luciferase assay

The *Gaussia* luciferase assay was performed as previously described by Wille *et al*. [17].

### Quantification of SiiE retention

Bacterial strains were diluted 1:31 in LB from O/N cultures and grown at 37°C for 3.5 h. Aliquots of 1 ml of bacterial culture were collected, cells pelleted and resuspended in 1 ml of sterile LB. After an additional washing step with sterile LB, optical density was measured and adjusted to OD_600_ of 1 in 500 μl of 3% PFA in PBS. After fixation of bacterial cells for 15 min at RT, cells were pelleted (10,000 x g; 5 min) and resuspended in 500 μl PBS. Five microliters of bacterial suspensions were spotted on a nitrocellulose membrane which has been pre-wetted with PBS and dried again before adding bacteria. After drying of the spots, membranes were blocked with 5% dry milk powder in TBS/T (TBS; 0.1% Tween20) for at least 30 min. For detection of SiiE on the bacterial surface, antiserum against the C-terminal moiety of SiiE [35] was diluted 1:10,000 in blocking solution and applied to the membrane. LPS was detected using antiserum against *Salmonella* O-antigen (Becton-Dickinson) at the same dilution. After incubation O/N at 4°C, membranes were washed thrice with TBS/T and bound primary antibodies were detected with anti-rabbit IRDye 800CW (LI-COR) at a dilution of 1:20,000 in PBS/T (PBS; 0.1% Tween20). Subsequently, membranes were incubated for 1 h at RT in the dark and washed thrice with PBS/T. Membranes were rinsed in PBS and signals were quantified using the Odyssey Imaging System (LI-COR Biotechnology).

### Quantification of secreted SiiE

Bacterial strains were diluted 1:31 in LB from an O/N culture and grown at 37°C for 6 h. The OD_600_ was measured. Aliquots of 2 ml of bacterial culture were taken and pelleted. Supernatants were filter sterilized (0.2 μm Millex filter units, Millipore). 200 μl of 100% TCA was added to 1.8 ml of filtered sterilized supernatant and incubated O/N at 4°C. The precipitated proteins were pelleted by centrifugation at 4°C for 45 min. After two washing steps with 1 ml ice-cold acetone (14,000 x g, 30 min, 4°C) the precipitate was air dried and afterwards resuspended in 25 μl PBS per OD_600_ of 1. Dot blot analysis was carried out as described before without detection of LPS.

### Determination of SiiE by Western blot analysis

For Western blot analysis, O/N cultures were diluted 1:31 in LB and grown for 3.5 h at 37°C. OD_600_ was measured, and 150 μl were pelleted in 2 min at 4°C at 16.000 x g. The pellet was resuspended in OD_600_ x 50 μl in 1 x SDS sample buffer and incubated at 100°C for 5 min. 15 μl of each sample were loaded onto 3–8% gradient gels (NuPage) and electrophoretically separated for 1 h at 150 V. Semi-dry blotting was performed using a 0.2 μm nitrocellulose membrane with 64 mA/blot for 4 h. After blocking with 5% milk/TBS/T, the membrane was incubated first with a primary antibody against SiiE and subsequently with a secondary HRP-conjugated antibody against rabbit IgG. The signals were determines using ECL reagent (ThermoScientific) and the ChemiDoc system (BioRad).

### Invasion assay

Invasion assay was performed as previous described by Wagner *et al*. [6]. Briefly, O/N cultures of *Salmonella* strains were diluted 1:31 in LB and grown for 3.5 h in test tubes with aeration in a roller drum. The cultures were diluted in MEM medium to obtain a multiplicity of infection (MOI) of 5 and this inoculum as added to the MDCK cells. After infection for 25 min cells were washed three times with PBS to remove non-internalized bacteria, and medium was replaced by medium containing 100 μg x ml^-1^ gentamicin to kill remaining extracellular bacteria. After incubation for 1 h, cells were washed again with PBS, lysed by addition of 0.5% sodium desoxychlate in PBS, and colony forming units were determined by plating serial dilutions of the lysates onto agar plates.

### Protein production of SiiE_Cterm_ variants for in vitro characterization

All SiiE_Cterm_ variants (p4033, p4034, p4462, p4463, S1 Table) were cloned into the pGEX-6P-1 vector (GE Healthcare) and expressed as GST fusion constructs in *E*. *coli* BL21 (DE3) (Novagen). The bacteria were chemically transformed with the expression plasmid and transformants selected on LB agar plates containing 100 μg x ml^-1^ ampicillin. The expression was done in terrific broth containing 100 μg x ml^-1^ ampicillin. A starter culture was inoculated with a single colony and grown over night at 37°C and 180 rpm. On the next day, the main expression cultures were inoculated to an OD_600_ of 0.08 and incubated at 37°C and 180 rpm. At OD_600_ 0.6 to 0.7 the temperature was reduced to 20°C and at OD_600_ 1.0 to 1.2 the protein expression was induced by adding 0.5 mM IPTG. After induction, the proteins were expressed for 20 h at 20°C and 180 rpm, the bacteria harvested by centrifugation and the pellets stored at -80°C until used for purification. Briefly, the GST-tagged proteins were captured by Glutathione Sepharose affinity column (GE Healthcare) using standard buffers given in the manual. The GST tag was cleaved by adding GST tagged HRV 3C protease at a mass ratio of 1 to 250 (protease-to-fusion protein) and the tag, undigested fusion proteins and the protease were extracted by a second Glutathione Sepharose purification. As a final purification step, the proteins were separated by size exclusion chromatography using a HiLoad 26/60 Superdex 200 pg column (GE Healthcare) and a buffer with 25 mM Tris-HCl, 150 mM NaCl, pH 8.0. No calcium was added to the buffers during the chromatographic purification. The protein was concentrated to 8 mg x ml^-1^, frozen in liquid nitrogen and stored at -80°C until use.

### Inductively coupled plasma—atom emission spectroscopy

The calcium content of the SiiE_Cterm_ variants was analysed by inductively coupled plasma—atom emission spectroscopy. All proteins were purified as described above, but without the second Glutathione Sepharose step after tag cleavage. The purified SiiE_Cterm_ proteins were concentrated and dialysed against the same batch of buffer (5 mM Tris-HCl, pH 8.0, ratio of sample-to-buffer volume 1:100). Calcium standard for ICP (Sigma-Aldrich) was diluted to 0.5, 5 and 50 μg x ml^-1^ with the same buffer. The ICP-AES analyses were performed using a Ciros CCD (Spectro Analytical Instruments GmbH). Three individual measurements of the same sample were conducted for each variant and the mean calculated.

### Limited proteolysis assays

Limited proteolysis was performed in order to investigate the influence of the mutations on the conformation and compactness of the proteins [36]. The proteolysis experiments were conducted at 20°C and 550 rpm in a benchtop shaker. The assay was done in a buffer containing 25 mM Tris-HCl, 150 mM NaCl, pH 8.0 and the SiiE protein concentration was adjusted to 1 mg x ml^-1^. 10 μg α-Chymotrypsin or 0.5 μg Proteinase K (Proti-Ace & Proti-Ace 2 Kit, Hampton Research, Aliso Viejo, USA) were added per mg of SiiE protein. Aliquots of 18 μl were taken prior (0 min), and at various time points after protease addition (1 min, 5 min, 15 min, 30 min, 1 h, 2 h, 4 h, 7 h, O/N). The samples were mixed with 6 μl 4 x SDS-PAGE loading buffer and boiled at 95°C for 5 min to stop the cleavage reaction. The heat-treated samples were briefly spun down and stored at -20°C. The 10 μl of each sample were analyzed by SDS-PAGE using 15% polyacrylamide gels. Gels were stained with Coomassie Blue.

### Circular dichroism spectroscopy

The conformation and stability of SiiE variants were probed using circular dichroism (CD) spectroscopy. All measurements were done with a Jasco J-815 spectropolarimeter (Jasco, Tokyo, Japan) using a cuvette with a 0.1 cm path length. CD spectra were recorded at 20°C in the far UV region between 185 and 260 nm in 10 mM potassium phosphate buffer, pH 8.0. Protein concentrations of 15 μM were used for SiiE_Cterm_ variants. The band width was set to 1.0 nm, the scan speed to 20 nm x min^−1^, data integration time to 1 sec, data pitch to 0.1 nm and sensitivity to standard. Each measurement was averaged across ten accumulations and the protein spectra corrected for the sample buffer.

The stability of the proteins was compared by thermal scanning analysis. Changes in the secondary structure composition were investigated between 20°C and 96°C by monitoring the CD signal at wavelengths of 222 nm for SiiE_Cterm_ proteins. A band width of 1.0 nm, data integration time of 8 sec, heat rate of 1°C x min^-1^, sampling rate of one data point per 0.2°C and standard sensitivity was used for all thermal scanning experiments. Conversion of the data to concentration- and length-independent mean residue weight (MRW) ellipticities [θ]_MRW_ was done as described previously [37]. The secondary structure analysis of the CD-spectra and of the Ig-domain structure of SiiE BIg domains 50 to 52 (PDB entry: 2YN5) was done with single spectrum analysis and the secondary structure and beta-sheet decomposition for PDB-structures tools of the BeStSel server, respectively (http://bestsel.elte.hu/ssfrompdb.php) [38]. The wavelength range between 190 nm and 250 nm of the CD spectra was used for estimation of the secondary structure content.

### Native PAGE

Native polyacrylamide gel electrophoresis (PAGE) was used to analyze the aggregation tendency of the SiiE variants. All protein samples were adjusted to 0.3125 mg x ml^-1^ and 20 μl samples of each protein were incubated at various temperatures (20, 30, 41.7, 50, 60, 70, 80 and 90°C) for 5 min, briefly spun down and chilled on ice for 1 min. 5 μl of 5 x native PAGE-buffer (0.25% (w/v) Bromophenol blue, 4.5% (w/v) sucrose) were added to each sample to achieve a final protein concentration of 0.25 mg x ml^-1^ and 15 μl (3.75 μg protein) were loaded per sample. Native PAGE was done using 7.5% native polyacrylamide gels and native PAGE running buffer (50 mM Tris, 384 mM glycine). The gel runs were done at 5 mA per gel for 5 h and 6–8°C in the cold room and gels were stained with Coomassie Blue. For the SDS-PAGE analysis, 4 μl of 4 x SDS sample buffer were added to 16 μl of each sample, the mixture boiled at 95°C for 5 min and briefly centrifuged. 10 μl of each of the samples were analyzed on 15% acrylamide gels. The gel runs were done at 200 V for 60 min.

### Bioinformatics

The crystal structure of SiiE wild-type BIg domains 50–52 (PDB code 2YN5, chain A) was used for all computational studies. Based on the wild-type system, three mutants were modelled that lack either the type I, the type II, or both types of Ca^2+^-binding sites. Mutants were generated by replacing the respective coordinating aspartate and glutamate residues by serine.

All systems were neutralized by adding an appropriate amount of sodium counter ions. Each system was placed in a periodic TIP3P water box [39] extending at least 12 Å in all directions from the solute. All simulations were done with Amber 14 [40] using the ff99SB force field [41]. Long-range electrostatics were calculated with the particle mesh Ewald (PME) approximation [42]. Shake was used to constrain hydrogen atoms during equilibration and simulation [43].

Minimization, equilibration and MD calculations were carried out with the pmemd module of AMBER. Minimizations were run for 10,000 steps and switched from steepest descent to conjugate gradient after 500 cycles. During equilibration the system was gradually heated from 30 K to 310 K in 60 ps with backbone restraints of 2.0 kcal/mol Å^2^ and then relaxed at 310 K with backbone restraints of 0.2 kcal/mol Å^2^ for another 20 ps. Two independent production runs of 300 ns were generated for each system using the weak-coupling algorithm [44] and a Berendsen barostat in an NPT ensemble.

For the SMD simulations ten restart files containing atomic coordinates and velocities were taken from wild-type MD simulation (at intervals of 10 ns). Ca^2+^ ions were pulled from type I and type II sites of SiiE individually by a harmonic potential with the spring energy constant of 50 kcal/mol Å^2^. The center of this potential was moved away from the center of mass (CoM) of the backbone atoms of one BIg domain with a constant velocity of 0.2 Å/ps. In this way the force was evenly distributed on the protein and no positional restraints had to be used.

## Supporting information

S1 Table Plasmids used in this study. (DOCX)

S2 Table Oligonucleotides used in this study. (DOCX)

S3 Table Synthetic DNA fragments used in this study. (DOCX)

S4 Table Estimated secondary structure content (%). (DOCX)

S1 Fig Secretion reporter assay. A) The Gaussia Luciferase (GLuc) converts its substrate coelenterazine to coelenteramide and photons. Emitted light can be detected as read out of the conversion. B) Assay for quantification of synthesis and secretion of GLuc-SiiE fusion proteins. Plasmids were introduced in *Salmonella* WT and Δ*siiF* strains. The Δ*siiF* strain is unable to secrete SiiE due to lacking the ATPase. O/N cultures were diluted 1:31 in LB containing 50 μg x ml^-1^ carbenicillin and grown for 6 h. Cells were pelleted and the filter-sterilized supernatant contains secreted GLuc-SiiE. The cell pellet was resuspended in assay buffer lysed with glass beads. This sample contains retained and cytosolic SiiE. C) Secretion reporters were constructed consisting of GLuc and the C-terminal moiety of SiiE, i.e. BIg47-53. Ca^2+^-binding sites were deleted by D/S exchanges of various extent as indicated by blue boxes. The number of deleted Ca^2+^-binding sites is indicated by Δ2, Δ4, Δ6 or Δ10. D) GLuc assay of various reporter fusions. GLuc activity is expressed as light counts per second (LCPS) standardized by the OD_600_ of the cultures. Means and standard deviations of triplicates are shown as percentage of the GLuc-SiiE_WT_ activity. One representative experiment of three replicates is shown.
(TIF)

S2 Fig Role of conserved tryptophan residues in BIg of SiiE. The W/F exchanges of conserved residue 74 were performed for single BIg domains 50, 51 or 52, for two BIg domains 50/51, 51/52 or 50/52, or for three BIg domains 50–52. A) Schematic overview of mutant *siiE* alleles. B) Synthesis of the mutant SiiE variants was tested by Western blot. C) Analyses of amounts of retained SiiE (C) and secreted SiiE (D) after 3.5 h and 6 h of subculture. E) SiiE-dependent invasion of polarized epithelial MDCK cells. Analyses of synthesis, surface retention and secretion and SiiE-dependent invasion of polarized cells were performed as described for Fig 2 of the main text.
(TIF)

S3 Fig Secretion of fusion proteins consisting of the C-terminal portion of SiiE (BIg50-BIg53), and MalE WT or mutant forms with altered folding kinetics. Synthesis and secretion of MalE-SiiE fusion proteins by *Salmonella* WT or Δ*siiF* strains was analyzed after 3.5 and 6 h of subculture. Western blots were performed with total cell fractions (pellet) and culture supernatants using antisera against SiiE.
(TIF)

S4 Fig The atom emission spectra of calcium standards and of SiiECterm variants with WT sequence and double mutant BIg48-52Δ8_type I + II_. Shown are emission spectra around the calcium line at 396.847 nm. The 0.5, 5, and 50 μg x ml^-1^ calcium standards are coloured in olive, light green and purple, respectively. The wild-type protein is coloured in red and the BIg48-52Δ8_type I + II_ mutant, the lowest curve, in dark green.
(TIF)

S5 Fig Thermal stability of SiiE. SiiE_Cterm_ variants were subjected to incubation at various temperatures as indicated, and samples are analyzed SDS-PAGE rather than by native PAGE as for Fig 6E. The molecular weights of the marker bands (M) are indicated.
(TIF)

S6 Fig Folding and compactness of BIg of SiiE probed by limited proteolysis. C-terminal portions of SiiE comprising BIg50 to the C-terminus (SiiE_Cterm_) were subjected to limited proteolysis by Chymotrypsin (Chym, left column) or Proteinase K (ProtK, right column). SiiE_Cterm_ WT (WT, A), mutant proteins with D/S exchange in type I and type II Ca^2+^-binding sites (B), type I-binding sites only (C), or type II-binding sites only (D) were analyzed. Proteins were incubated with proteases for various time intervals as indicated (‘, minutes; h, hours; oN, overnight), reactions were stopped and degradation was analyzed by SDS-PAGE. Samples without protease (-ProtK, -Chym) were incubated and analyzed accordingly as negative controls. The molecular weights of the individual marker bands (M) are indicated.
(TIF)

S7 Fig Influence of single D/S exchanges on SiiE function. Mutations of chromosomal *siiE* were generated resulting in single D/S exchanges in BIg51/52, or D/S exchanges of type I or type II Ca^2+^-binding sites. A) Schematic overview of mutant *siiE* alleles. B) Synthesis of the mutant SiiE variants was tested by Western blot. C) Analyses of amounts of retained SiiE (C) and secreted SiiE (D) after 3.5 h and 6 h of subculture. E) SiiE-dependent invasion of polarized epithelial MDCK cells. Analyses of synthesis, surface retention and secretion and SiiE-dependent invasion of polarized cells were performed as described for Fig 2 of the main text.
(TIF)
