# Supplementary material for: Structural and functional dissection reveals distinct roles of Ca2+-binding sites in the giant adhesin SiiE of Salmonella enterica
Source: PLoS Pathog. 2017 May 30;13(5):e1006418. doi: 10.1371/journal.ppat.1006418 (PMC5466336; doi:10.1371/journal.ppat.1006418)
Supplement: S1 Table — (DOCX) [file ppat.1006418.s001.docx]

Table S 1. Plasmids used in this study

| **Designation** | **relevant characteristics** | **reference** |
| --- | --- | --- |
| pWRG454 | pWSK29, *P_siiA_* GLuc_M43LM110L_::*siiE* BIg50-53 | this study |
| pWRG717 | pSIM-I *Sce*I *aph* | RGG, unpublished |
| pWRG730 | Tet-inducible expression of λ Red recombinase, ts, Amp^R^ | RGG, unpublished |
| p3355 | pGEX-6P-1, GST::*siiE*_6-403_::*siiE*_5052-5559_ | this study |
| p3855 | pJET1.2, *siiE* BIg52_D-S_ | this study |
| p3902 | pWSK29, *P_siiA_* GLuc_M43LM110L_::*siiE* BIg50-53_BIg52D-S_ | this study |
| p3936 | pJET1.2, *siiE* BIg47-49_half D-S_ | this study |
| p3937 | pJET1.2, *siiE* BIg49_half_-52_D-S_ | this study |
| p3940 | pWSK29, *P_siiA_* GLuc_M43L M110L_::*siiE* BIg49_half_-52_D/S_ | this study |
| p3941 | pWSK29, *P_siiA_* GLuc_M43L M110L_::s*iiE* BIg47-53_D/S_ | this study |
| p3970 | pWSK29, *P_siiA_* GLuc_M43L M110L_::s*iiE* BIg47-53 | this study |
| p3973 | pWSK29, *P_siiA_* GLuc_M43L M110L_::s*iiE* BIg47_D16S D24S D43S_-BIg53_BIg51-52 D/S_ | this study |
| p3975 | pWSK29, *P_siiA_* GLuc_M43L M110L_::s*iiE* BIg47_D-S_-Ig53_BIg51-52 D-S_ | this study |
| p4013 | pWSK29, *P_siiA_* GLuc_M43L M110L_::s*iiE* BIg47-53_BIg51 D117S_ | this study |
| p4014 | pWSK29, *P_siiA_* GLuc_M43L M110L_::*siiE* BIg47-53_BIg52 D16S_ | this study |
| p4015 | pWSK29, *P_siiA_* GLuc_M43L M110L_::*siiE* BIg47-53_BIg52 D24S_ | this study |
| p4016 | pWSK29, *P_siiA_* GLuc_M43LM110L_::s*iiE* BIg47-53_BIg52 D43S_ | this study |
| p4017 | pWSK29, *P_siiA_* GLuc_M43LM110L_::s*iiE* BIg47-53_BIg52 D97S_ | this study |
| p4029 | pWSK29, *P_siiA_* GLuc_M43LM110L_::s*iiE* BIg47-53_BIg52 D16S_ _D24S_ | this study |
| p4030 | pWSK29, *P_siiA_* GLuc_M43LM110L_::s*iiE* BIg47-53_BIg51 D117S Ig52 D43S D96S_ | this study |
| p4033 | pGEX-6P-1, GST::*siiE* BIg48-53 | this study |
| p4034 | pGEX-6P-1, GST::*siiE* BIg48-53_BIg48-52D/S_ | this study |
| p4035 | pGEX-6P-1, GST::*siiE* BIg1-5::*siiE* BIg48-53 | this study |
| p4053 | pWSK29, *P_siiA_* GLuc_M43LM110L_::*siiE* BIg47-53_BIg52D-S_ | this study |
| p4082 | pGEX-6P-1, GST::*siiE*_6-403_::*siiE_5052-5559_* _BIg50 W74F_ | this study |
| p4083 | pGEX-6P-1, GST::*siiE*_6-403_::*siiE_5052-5559_* _BIg51 W74F_ | this study |
| p4084 | pGEX-6P-1, GST::*siiE*_6-403_::*siiE_5052-5559_* _BIg52 W74F_ | this study |
| p4136 | pGEX-6P-1, GST::*siiE*_6-403_::*siiE_5052-5559_* _BIg50/51 W74F_ | this study |
| p4137 | pGEX-6P-1, GST::*siiE*_6-403_::*siiE*_5052-5559_ _BIg50/52 W74F_ | this study |
| p4138 | pGEX-6P-1, GST::*siiE*_6-403_::*siiE*_5052-5559 BIg51/52 W74F_ | this study |
| p4139 | pGEX-6P-1, GST::*siiE*_6-403_::*siiE*_5052-5559 BIg50-52 W74F_ | this study |
| p4140 | pWSK29, *P_siiA_ malE*_WT_::*siiE* BIg50-53 | this study |
| p4141 | pWSK29, *P_siiA_ malE*_V8G_::*siiE* BIg50-53 | this study |
| p4142 | pWSK29, *P_siiA_ malE*_V276G_::*siiE* BIg50-53 | this study |
| p4143 | pWSK29, *P_siiA_ malE*_Y283D_::*siiE* BIg50-53 | this study |
| p4178 | pJET1.2, *siiE* BIg39-41_BIg40 D/S_ | this study |
| p4198 | pJET1.2, *siiE* BIg47-52 _type II D/S_ | this study |
| p4200 | pJET1.2, *siiE* BIg1-5 _D/S_ | this study |
| p4201 | pJET1.2, *siiE* BIg47-52 _type I D/S_ | this study |
| p4217 | pJET1.2, *siiE* BIg1-5 _Scramble_ | this study |
| p4311 | pWSK29, *P_siiA_ siiE* BIg1-5::*siiE* BIg47-53_WT_ | this study |
| p4312 | pWSK29, *P_siiA_ siiE* BIg1-5::*siiE* BIg47-53_BIg47-52 D/S_ | this study |
| p4313 | pWSK29, *P_siiA_ siiE* BIg1-5::*siiE* BIg47-53_BIg47-52 type I D/S_ | this study |
| p4314 | pWSK29, *P_siiA_ siiE* BIg1-5::*siiE* BIg47-53_BIg47-52 type II D/S_ | this study |
| p4423 | pJET1.2, *siiE* BIg1-5 _type I D/S_ | this study |
| p4424 | pJET1.2, *siiE* BIg1-5 _type II D/S_ | this study |
| p4425 | pJET1.2, *siiE* BIg47-52 _Scramble_ | this study |
| p4459 | pGEX-6P-1, GST::*siiE* BIg1-5_D/S_::*siiE* BIg48-53 | this study |
| p4460 | pGEX-6P-1, GST::*siiE* BIg1-5_type I D/S_::*siiE* BIg48-53 | this study |
| p4461 | pGEX-6P-1, GST::*siiE* BIg1-5_type II D/S_::*siiE* BIg48-53 | this study |
| p4462 | pGEX-6P-1, GST::*siiE* BIg1-5::*siiE* BIg48-53 BIg48-52 _type II D/S_ | this study |
| p4463 | pGEX-6P-1, GST::*siiE* BIg1-5::*siiE* BIg48-53BIg48-52 _type I D/S_ | this study |
