# Supplementary material for: Structural and functional dissection reveals distinct roles of Ca2+-binding sites in the giant adhesin SiiE of Salmonella enterica
Source: PLoS Pathog. 2017 May 30;13(5):e1006418. doi: 10.1371/journal.ppat.1006418 (PMC5466336; doi:10.1371/journal.ppat.1006418)
Supplement: S2 Table — (DOCX) [file ppat.1006418.s002.docx]

Table S 2. Oligonucleotides used in this study

| Designation | Sequence (5’ - 3’) |
| --- | --- |

cloning

| SiiE_Ig47-GA-For | ataagcttagtacgtttatCGATAATCCTGCTATGGTG |
| --- | --- |
| SiiE_Ig50-GA-Rev | ataacgggaacctctttcatGATATCGAGAGGGACATCATCACTCC |
| SiiE_Ig48-GA-For | ttctatcggtggacaaaCGTTAGCAGAGATGACCG |
| SiiE-Ins-Rev | accgctagcgaagcagattg |
| SiiE_Ig52-GA2-Rev | agctaaccgtcaccgctagcgaagcagattgctgtgaa |
| SiiE_Ig52-GA2-For | ACTGTCACGATCAGCAGCACGCTGACGGTGCCGGAG |
| SiiE_Ig50-GA-For | aACGCCGCCAAATGCTCCG |
| SiiE_Ig51-GA2-Rev | cgcaatctccggtaccgtcagcgtgctgtcgatcgt |
| SiiE mut D5256S For | TACCCATTACTGTCACGATCAGCAGCACGCTGACG |
| SiiE_D5256S_Q5-Rev | ACGAGTTTTTCTGATTAC |
| SiiE mut D5271S For | TTGGCAGCAGGTGAATCGAATGGCGCTTCAGACAG |
| SiiE_D5271S_Q5-Rev | CGCAATCTCCGGCACCGTC |
| SiiE mut D5278S Hyp188II For | TTGGCAGCAGGTGAATCGAATGGCGCTTCAGACAG |
| SiiE_D5278S_Q5-Rev | CATTGTCTTCACCTGCTG |
| SiiE mut D5269S For | CTGCAGCATATTGATGCTAGCGTGACCGGGGTGAC |
| SiiE_D5296S_Q5-Rev | CGTGAACTTAGGCTGGGTG |
| SiiE mut D5334S For | GCTGAGCGTGACGGTGGTGAGTCGAGCGGGGAAT |
| SiiE_D5334S_Q5-Rev | GTGTAGTTACCGTCATTC |
| SiiE14632-For-BamHI | ctcggatcctttaatacgacgcctgtcgcc |
| GST-SiiE-C-Rev-SalI | agtgtcgacttatgcgtgttcttcttgattatctac |
| SiiE BIg 1-5 For | GAAAATAAGCTCGATGCCGAGTCTGTTAAAGAGCCGCTTAAAGTCACA |
| pGex SiiE N-Rev | TTTAACAGACTCGGCATCGAGCTTATTTTCC |
| pGex SiiE N-For | CATATCAAGGTATTTACCAGCGAGCTTGATGA |
| SiiE BIg 1-5 Rev | TCAAGCTCGCTGGTAAATACCTTGATATGAGTTTGAATAGTAAAAGAATATTTAACAGTA |
| pGex SiiE C-For | GTGACAGCCTATAGTATTACATTGTTAAACGCTGACTCTGG |
| SiiE BIg48-52 Rev | GTTTAACAATGTAATACTATAGGCTGTCACTGAGTCAACCGTCACCGCTA |
| pGex SiiE C-Rev | GGATCCCAGGGGCCCCTGGAACAGAACTTC |
| SiiE BIg48-52 For | GAAGTTCTGTTCCAGGGGCCCCTGGGATCCTTTAATACGACGCCTGTCGC |
| pWRG454_GA_For | ACGCCGCCAAATGCTCCGGTCGTAACGTAT |
| pWRG454_GA_Rev | TTCTAGAGTCTCCTGATATTACATTGTGAATAAAATGTTTTTGTGGATTAGAAAGG |
| MalE_GA_For | TTCACAATGTAATATCAGGAGACTCTAGAAATGAAAATCGAAGAAGGTAAA |
| MalE_GA_Rev | ATACGTTACGACCGGAGCATTTGGCGGCGTCGAGCTCGAATTAGTCTG |
| p4031_GA_For | TAAGCGGCCGCCACCGCGGTGGAGCTCCAG |
| MiniSiiE_GA-Rev | CTGGAGCTCCACCGCGGTGGCGGCCGCTTATTATGCGTGTTCTTCTTGAT |
| MiniSiiE_GA-For | TCTACTGTTAAATATTCTTTTACTATTCAAACTAGTACGTTTATCGATAATCCT |
| p4031_GA_Rev | AGTTTGAATAGTAAAAGAATATTTAACAGTAGAGGATTTATTGCCGGCGGCATCCTGAGAAATGATTTCAATATCATAGGC |

Mutagenesis

| SiiE_Ig47-52-Del-I-SceI-aph-For | TACGGCAGAATCGCCTCGCTTACTCGTCACGATAGATACCAGGGTTTTCCCAGTCACGAC |
| --- | --- |
| SiiE_Ig47-52-Del-I-SceI-aph-Rev | TCGCATCGTCATGCTGGCTATCCGCTGTTACCGTCACCGTTGCTTCCGGCTCGTATGTTG |
| SiiE-Ig47-52-RepI-For | TACGGCAGAATCGCCTCGCTTACTCGTCACGATAGATACCAGTACGTTTATCGATAATCC |
| SiiE-Ig47-52-RepI-Rev | TGTACGAAGATGCGTAGCGC |
| SiiE Ig1 I-SceI-aph for | TCCCAATGCCACGGTTATTATTAAAATTAATGGTATTGCTAGGGTTTTCCCAGTCACGAC |
| SiiE Ig2 I-SceI-aph rev | TACTATCATCCAGCTCAATCGTCGGCTTTTCTGGAGCAATTGCTTCCGGCTCGTATGTTG |
| SiiE Ig39 I-SceI-aph for | GAAACTGGTGATTACCATTGTTGACGATAAGTCAGGTCGGAGGGTTTTCCCAGTCACGAC |
| SiiE Ig40 I-SceI-aph rev | CACTATCGCTCTCCTCGCTGAGCTTTATGGTTGGAGGCGTTGCTTCCGGCTCGTATGTTG |
| SiiE15475-I-SceI-aph-For | TGCTATTGGTAACCGGAGTGATGATGTCCCTCTCGATATCAGGGTTTTCCCAGTCACGAC |
| SiiE 708-Red717-For | TAAAAATGAGGAAAATAAGCTCGATGCCGAGTCTGTTAAAAGGGTTTTCCCAGTCACGAC |
| SiiE 2173-Red717-Rev | CATCACTGGCATCGAGTTCCGCTTTTGGAGGTACAACTTCTGCTTCCGGCTCGTATGTTG |
| SiiE 15474-Red717-Rev | CTGAGTCTGGAGATAATGAAATAACGGGAACCTCTTTCATTGCTTCCGGCTCGTATGTTG |
| SiiEDel15232-15474-For | AAATCGTTGGGAAGATGTGCGTGAAATATTTATTGACCGAATGAAAGAGGTTCCCGTTATTTCATTATCTCCAGACTCAG |

Check PCR

| Gluc-Check-For | GCATTGGCGAAGCGATTGTG |
| --- | --- |
| SiiE-16058-Rev | AGCGGAACTTCGAATATTGC |
| SiiE-15331-Check-For | AAAAATGCCGATGGCGTTTGGTTCTTTACG |
| SiiE-733-For | cttgcggccgagagtaacag |
| SiiE-1611-Rev | CGCTATCATTCGTCGTTTCA |
| SiiE-12088-For | accggaaattccaatagcgataacc |
| SiiE-12704-Rev | taggtaacgtgctcccaatcaggg |
| pGEX 3`-seq | ccgggagctgcatgtgtcagagg |
| pGEX 5`-seq | gggctggcaagccacgtttggtg |
| SiiE-15192-For | AAATCGTTGGGAAGATGTGC |
| siiE15846-check-Rev | ATGCTGCAGCGTGAACTTAGGCTGGGTGTG |
| k1-red-del | cagtcatagccgaatagcct |
| k2-red-del | cggtgccctgaatgaactgc |
| SiiE-2264-Rev | CCTATCCGCTGTTCCCAGTA |
| SiiE-578-For | AAGCAACGCAGGCTTCTAAA |
| SiiE Check 430 for | CGTTGATGTCGATAAAGATG |
