# Supplementary material for: Structural and functional dissection reveals distinct roles of Ca2+-binding sites in the giant adhesin SiiE of Salmonella enterica
Source: PLoS Pathog. 2017 May 30;13(5):e1006418. doi: 10.1371/journal.ppat.1006418 (PMC5466336; doi:10.1371/journal.ppat.1006418)
Supplement: S3 Table — (DOCX) [file ppat.1006418.s003.docx]

Table S 3. Synthetic DNA fragments used in this study

| SiiE-BIg52__5D-S_ | TTACTCGAGTTCTTTACGCCAGGTACACCGTTAGCTGATGGTTCCTATACGATATCGGTAATCGCAAGCGATGCCGCGGGTAATCAGAAAAACTCGTTACCCATTACTGTCACGATCAGCAGCACGCTGACGGTGCCGGAGATTGCGTTGGCAGCAGGTGAATCGAATGGCGCTTCAGACAGCAGCAACGTGACGAATCACACCCAGCCTAAGTTCACGCTGCAGCATATTGATGCTAGTGTGACCGGGGTGACCGTAAACGTGACGCATAATGGCGTGACAGACATCTATCAGGCGACGCAAGGCGCGGATGGCTGGACCTTCACGCCGCCAGCCGCCTGGAATGACGGTAACTACACGCTGAGCGTGACGGTGGTGAGTCGCGCGGGGAATTCACAGCAATCTGCTTCGCTAGCGGTGACGGTTGACTCAACGGTGACGGTAACAGCGGATAGCCAGCATGACGATGCGAGCGATGACGCCACGGCAACAGCGGTTACTCCACCGGAGTCTGAAACAGTGAATGCCGAAAGCGCTACGCATCTTCGTACAGAGCCGTCTGCGGCGGAAGAAAGCGTGGTGAAGCTCGAGAGG |
| --- | --- |
| SiiE-BIg47-49_half D-S_ | ATTAAGCTTAGTACGTTTATCGATAATCCTGCTATGGTGGCAGGTTCTTCGAATGGTATTTTCAGTAATAGTAGTATAACGAGTCAGACCCGGCCTACGTTTAGTATTTTTGGAAGTATGAACCAGAGTGTTCAGATTTTCATTGATGGAGTGCTAGTCGATACGATCACGGTGACCGACAGAAATCAAGTTTATCGACCTGAGTCACCGTTGGGCGATGGTTCCCATAGCATTTATTATGTTATCACCAGTAAAGCAGGCAACACGGCTACCTCGAAAACGCTAAACTTTACTATCAGCACCTTTAATACGACGCCTGTCGCCATTGATTCTATCGGTGGACAAACGTTAGCAGAGATGACCGGTAGTTCGGGCAAAATATATATAACGGACACGACGCGTAACTTATTGTTTAGTGGCAGTGCCAGTCCCAATAGCAAAATAGAAATCATCATTAATGGCTTAAATGTGGGGGAAGTTTGGGTTAATGAAAAAGGCCACTGGCAGATGCCGGTGAACCCGCTTTATTTCACAGAAGGCCAACTGGATATCACTGTTAAATCTACGAGTCGTGCTGGTAACGTAAATCAGGAAAAGTATTCCATATGGGTTAGCACGCATATCAAGGTATTTACCAGCGAGCTTGATTCGAATAAATCATCATCGAAAACGAGTTGGTGGAGTAATAGCGATCTCATTACCATGCGAGGCACGGGTAGTATTGGCGCTACGGTATCATTAATCGTGGCTGGCGTCACGCTGGCAACTGCTGTTGTGGCGGCAACAGGACGATGGGAATTATCAACAGACAAGCTTCCA |
| SiiE-BIg49_half_-52_D-S_ | GACAAGCTTCCAGAAGGGACTTACGATATTAGTTTGGTCATTGAAAGTAGCGCCGGAAATCGTTGGGAAGATGTGCGTGAAATATTTATTAGCCGAACGCCGCCAAATGCTCCGGTCGTAACGTATTCAGATATTGTCAACGATCTAATTATTATGCAGGGGACGGCGGAAGCCAAATCTCAGCTAATAATAACCGATAGTGAGGGGAATACTTATACGTTAACCGTTCCTGATAATGGTAAATGGAGTATGGCTATCCCGTATCCATCAGAAGGGAAGTTTACCATTACGAGTGTGAGTGCTATTGGTAACCGGAGTGATGATGTCCCTCTCGATATCATGAAAGAGGTTCCCGTTATTTCATTATCTCCAGACTCATCGAGTGGTACGGTGGGCAGTAATATTACGCGAGATAAGCAACCTACCTTTATTATCGGGAATCTGGAAAGCAGTGTTGTGGTCGTTCAGGTCGATATCAATGGGACCGTATATAATGCTGAAAAAAATGCCGATGGCGTTTGGTTCTTTACGCCAGGTACACCGTTAGCTGATGGTTCCTATACGATATCGGTAATCGCAAGCAGTGCCGCGGGTAATCAGAAAAACTCGTTACCCATTACTGTCACGATCAGCAGCACGCTGACGGTACCGGAGATTGCGTTGGCAGCAGGTGAATCGAATGGCGCTTCAGACAGCAGTAACGTGACGAATCACACCCAGCCTAAGTTCACGCTGCAGCATATTGATGCTAGTGTGACCGGGGTGACCGTAAACGTGACGCATAATGGCGTGACAGACATCTATCAGGCGACGCAAGGCGCGGATGGCTGGACCTTCACGCCGCCAGCCGCCTGGAATGACGGTAACTACACGCTGAGCGTGACGGTGGTGAGTCGCGCGGGGAATTCACAGCAATCTGCTTCGCTAGCGGTGACGGTTAGCTCA |
| SiiE-BIg1-3BIg2D-S | GAGCCGCTTAAAGTCACATTAGCGCTTGCGGCCGAGAGTAACAGCGGTAGCAAAGATGATAGTATAACTAATTTTACCAAACCTCAGTTTGTAGGTAGCACTGCTCCCAATGCCACGGTTATTATTAAAATTAATGGTATTGCTGTCGGTCAGGCTGTAGCGGATAGTTTGGGTAACTTCACCTTTACAGCGCCTGAAACATTGACTGATGGAACATATAATCTGGAGGCAGAGGCCAAGACTGCTGATGGGAGCGGTAGCGCCAAACTTGTCATTACTATCTCGTCCGTTACCGATAAACCAACATTTGAACTTTCGCCTTCCAGTAGTGTGTCCGGTCATAAGGGCTTAACGCCGACCTTGACGCCTTCAATTGTTGGTACGGCGAGTGAGAATGCTAAGGTTGACATTTATGTAGATAATAAACTGGTTGCCAGCGTTGATGTCGATAAAGATGGAAACTGGAGTTATGAATTTAAGGATAATGAATTATCTGAGGGCGAAAATAGTATAAAAGTCGTTGCTGTATCTAAAGCAGGTAATAAAAACGAAACGACGGATAGTATCATAACCGACACCATTGCTCCAGAAAAGCCGACGATTGAGCTGGATGATAGTAGTGATTCCGGCATTAAAAATGACAACATTACAAATAGCACCCTGCCAACATTTATTGGTGTGGCGGAACCCGGTTCTACAGTCTCTATTTATCTTGGACTTAAACATCTTGGTGAGGTCATTGTTGCTAAAGATGGGACATGGAGCTATACGCTTACTACGCCGCTCAAGGATGGCGAATACAATATAACAGCAACAGCTACTGATATTGCCGGGCATACCTCAGCGACGGCAAATCTGCCTTTTACTATTGATACA |
| SiiE-BIg39-41_BIg40D-S_ | ACGACAAGCGTCAGTGTCAGAATGGAGCCAGCGTCTGATACCGGAAATTCCAATAGCGATAACCTTACGAATAAGCAAAATCCCAAATTCGAAGGTACTGCAGAGCCCAATGCGAAACTGGTGATTACCATTGTTGACGATAAGTCAGGTCGGGAGGTTTTAAAACAAACGATTACGGTTGGCGCTGATGGCAACTGGAGTGTGACGCCGAATATACTGCCGGATGGCATGTATACCATCAACGTCGTCGCAACAGATGTCGCGGGAAATACTGCGCAAACGCAGGAAAGATTCACTATCTCGACGGTTACGATCGATCCCACCATTCGCCTTTCGTCACCATCTATTGATTCCCAGCATGAAGCAACCAGCCTGCGTCCTGAGTTCAAAGGGTTTGCCTCGGCGTTCTCGACGATTATGATTCAGTGGGATGGGAAAGTGGTCGGCTCGGCAAACGCCAATGCGAATGGCGAATGGAGTTGGACGCCGCCATCAGTATTAGCGCCAGGCTCCTATGTTGTGAGCATTGTTGCCAAAAGCAAAGCGGGTAATGAATCGTCGCAGGTCGACTTTCCTGTCGTAATACCTGTTATTGATGTCACGCCTCCAACCATAAAGCTCAGCGAGGAGAGCGATAGTGGCGCCTTAGGAGACTTTACCACGAATAATAAAACGCCGACCCTGATTGGGAGCACGTTACCTAATACGATTGTGAGTATTTATGTGGATGGCGTGAAGGTCGGCGAGGCGACAGCGGATACAGCGGGTCGATATACTTTCCAGTTATCGGAAATGAAAGATGGCCATTATGTCGTCCAGGTGGGTATCGTCAACCCTCGCGATAATAGCGAACTGCGTTCTACCGCCGTTGATGTCACTATCGATACC |
