# Supplementary material for: Structural and functional dissection reveals distinct roles of Ca2+-binding sites in the giant adhesin SiiE of Salmonella enterica
Source: PLoS Pathog. 2017 May 30;13(5):e1006418. doi: 10.1371/journal.ppat.1006418 (PMC5466336; doi:10.1371/journal.ppat.1006418)
Supplement: S4 Table — (DOCX) [file ppat.1006418.s004.docx]

Table S 4. Estimated secondary structure content (%)

|  | **2YN5** | **WT** | **BIg48-52**  **Δ8 _type I + II_** | **BIg48-52**  **Δ4 _type I_** | **BIg48-52**  **Δ4 _type II_** |
| --- | --- | --- | --- | --- | --- |
| **Helix** | 0.0 | 0.0 | 0.0 | 0.0 | 2.3 |
| **Antiparallel** | 47.2 | 46.5 | 43.3 | 44.9 | 41.4 |
| **Parallel** | 2.1 | 2.5 | 5.1 | 5.7 | 6.2 |
| **Turn** | 11.5 | 10.3 | 10.5 | 9.5 | 9.7 |
| **Others** | 39.2 | 40.7 | 41.1 | 39.9 | 40.4 |
|  |  |  |  |  |  |
| **RMSD^1^** | ---- | 0.2413 | 0.2464 | 0.1755 | 0.1937 |
| **NRMSD^2^** | ---- | 0.11138 | 0.10565 | 0.07218 | 0.07747 |

^1^ Root mean squared deviation [M^‑1^cm^‑1^] (Calculated after automatic conversion to Delta epsilon Δε by the BeStSel server)

^2^ Normalized root mean squared deviation
