# Supplementary figures and images for: Structural and functional dissection reveals distinct roles of Ca2+-binding sites in the giant adhesin SiiE of Salmonella enterica
Source: PLoS Pathog. 2017 May 30;13(5):e1006418. doi: 10.1371/journal.ppat.1006418 (PMC5466336; doi:10.1371/journal.ppat.1006418)

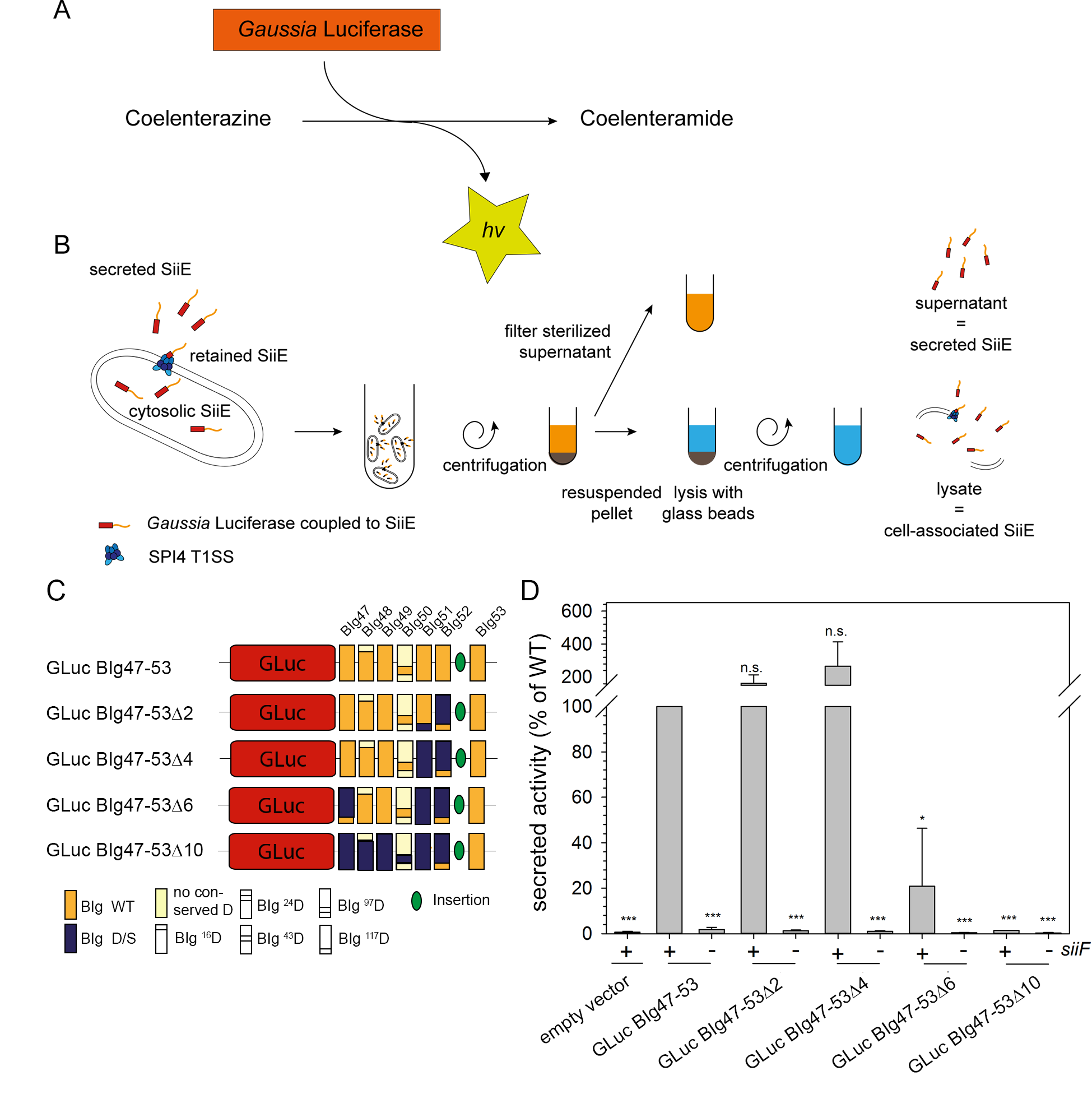

Supplement: S1 Fig — A) The Gaussia Luciferase (GLuc) converts its substrate coelenterazine to coelenteramide and photons. Emitted light can be detected as read out of the conversion. B) Assay for quantification of synthesis and secretion of GLuc-SiiE fusion proteins. Plasmids were introduced in Salmonella WT and ΔsiiF strains. The ΔsiiF strain is unable to secrete SiiE due to lacking the ATPase. O/N cultures were diluted 1:31 in LB containing 50 μg x ml-1 carbenicillin and grown for 6 h. Cells were pelleted and the filter-sterilized supernatant contains secreted GLuc-SiiE. The cell pellet was resuspended in assay buffer lysed with glass beads. This sample contains retained and cytosolic SiiE. C) Secretion reporters were constructed consisting of GLuc and the C-terminal moiety of SiiE, i.e. BIg47-53. Ca2+-binding sites were deleted by D/S exchanges of various extent as indicated by blue boxes. The number of deleted Ca2+-binding sites is indicated by Δ2, Δ4, Δ6 or Δ10. D) GLuc assay of various reporter fusions. GLuc activity is expressed as light counts per second (LCPS) standardized by the OD600 of the cultures. Means and standard deviations of triplicates are shown as percentage of the GLuc-SiiEWT activity. One representative experiment of three replicates is shown. (TIF) [file ppat.1006418.s005.tif]

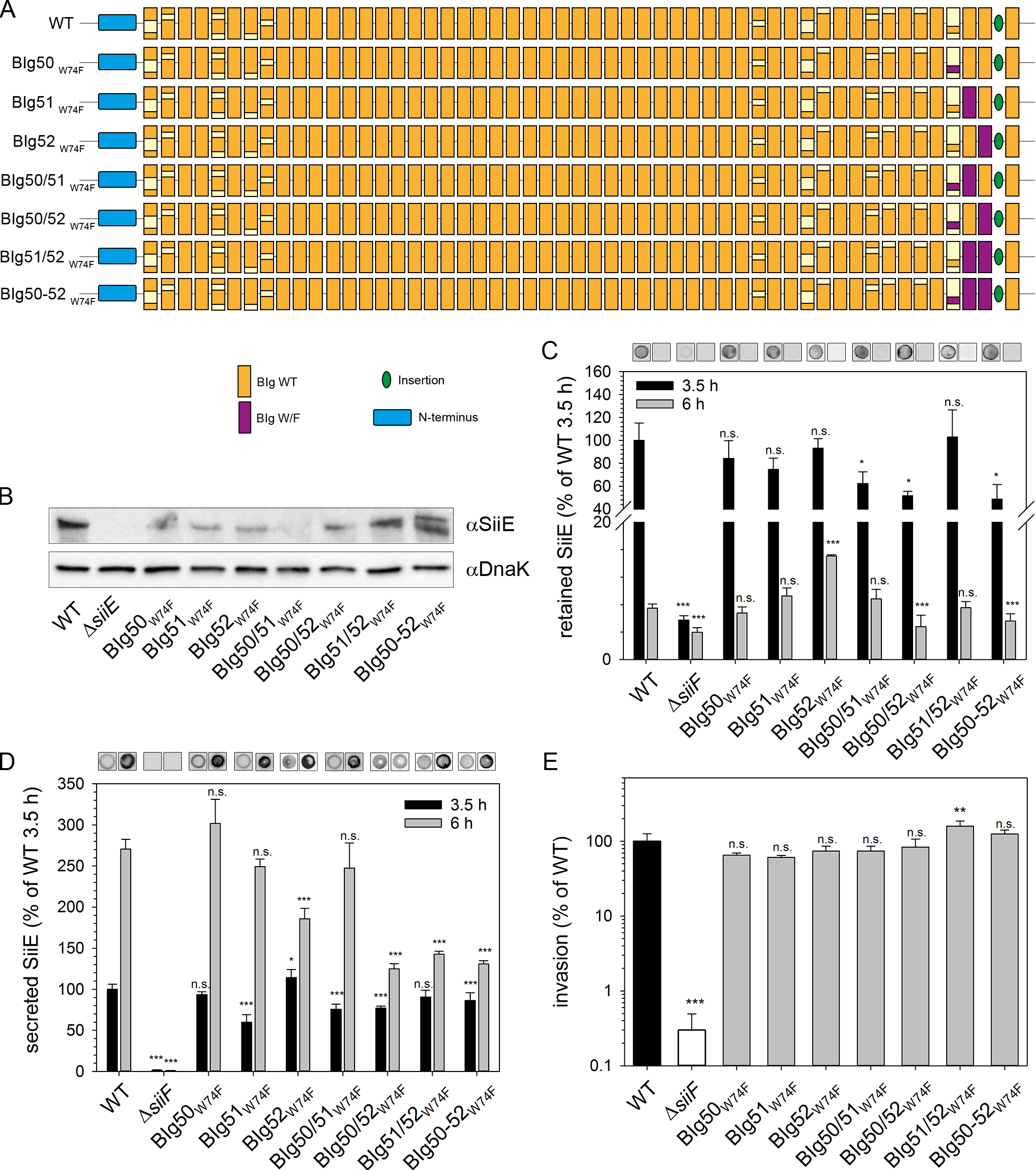

Supplement: S2 Fig — The W/F exchanges of conserved residue 74 were performed for single BIg domains 50, 51 or 52, for two BIg domains 50/51, 51/52 or 50/52, or for three BIg domains 50–52. A) Schematic overview of mutant siiE alleles. B) Synthesis of the mutant SiiE variants was tested by Western blot. C) Analyses of amounts of retained SiiE (C) and secreted SiiE (D) after 3.5 h and 6 h of subculture. E) SiiE-dependent invasion of polarized epithelial MDCK cells. Analyses of synthesis, surface retention and secretion and SiiE-dependent invasion of polarized cells were performed as described for Fig 2 of the main text. (TIF) [file ppat.1006418.s006.tif]

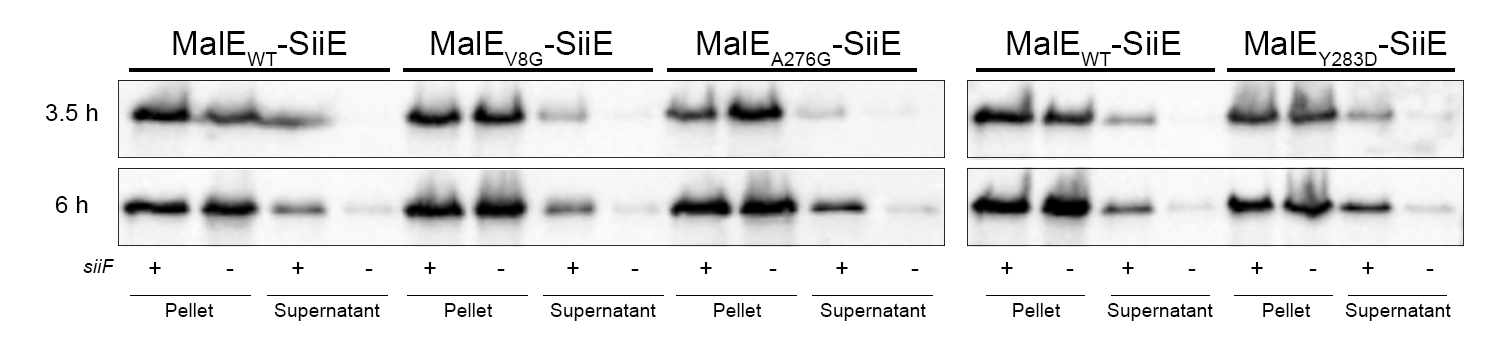

Supplement: S3 Fig — Synthesis and secretion of MalE-SiiE fusion proteins by Salmonella WT or ΔsiiF strains was analyzed after 3.5 and 6 h of subculture. Western blots were performed with total cell fractions (pellet) and culture supernatants using antisera against SiiE. (TIF) [file ppat.1006418.s007.tif]

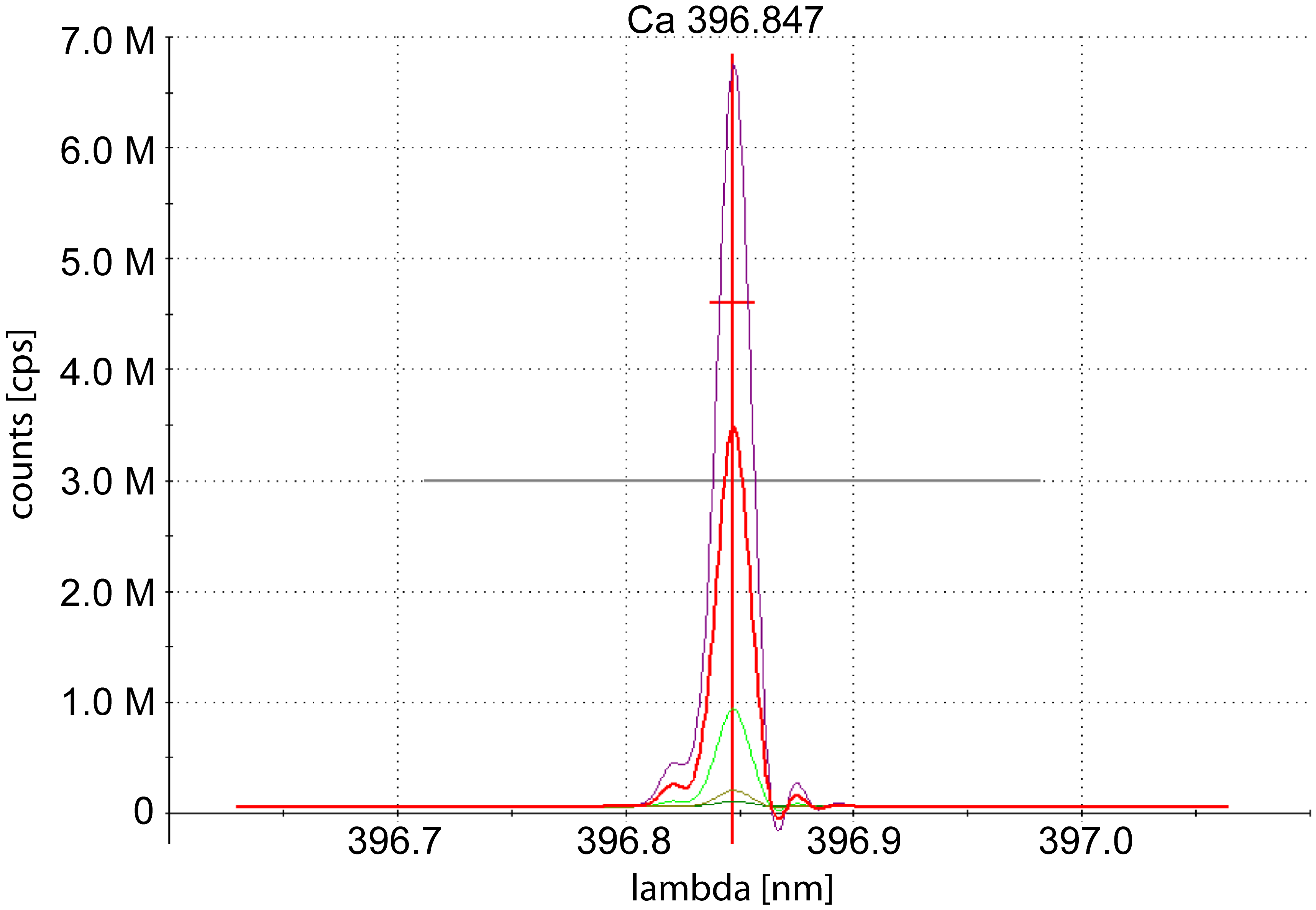

Supplement: S4 Fig — Shown are emission spectra around the calcium line at 396.847 nm. The 0.5, 5, and 50 μg x ml-1 calcium standards are coloured in olive, light green and purple, respectively. The wild-type protein is coloured in red and the BIg48-52Δ8type I + II mutant, the lowest curve, in dark green. (TIF) [file ppat.1006418.s008.tif]

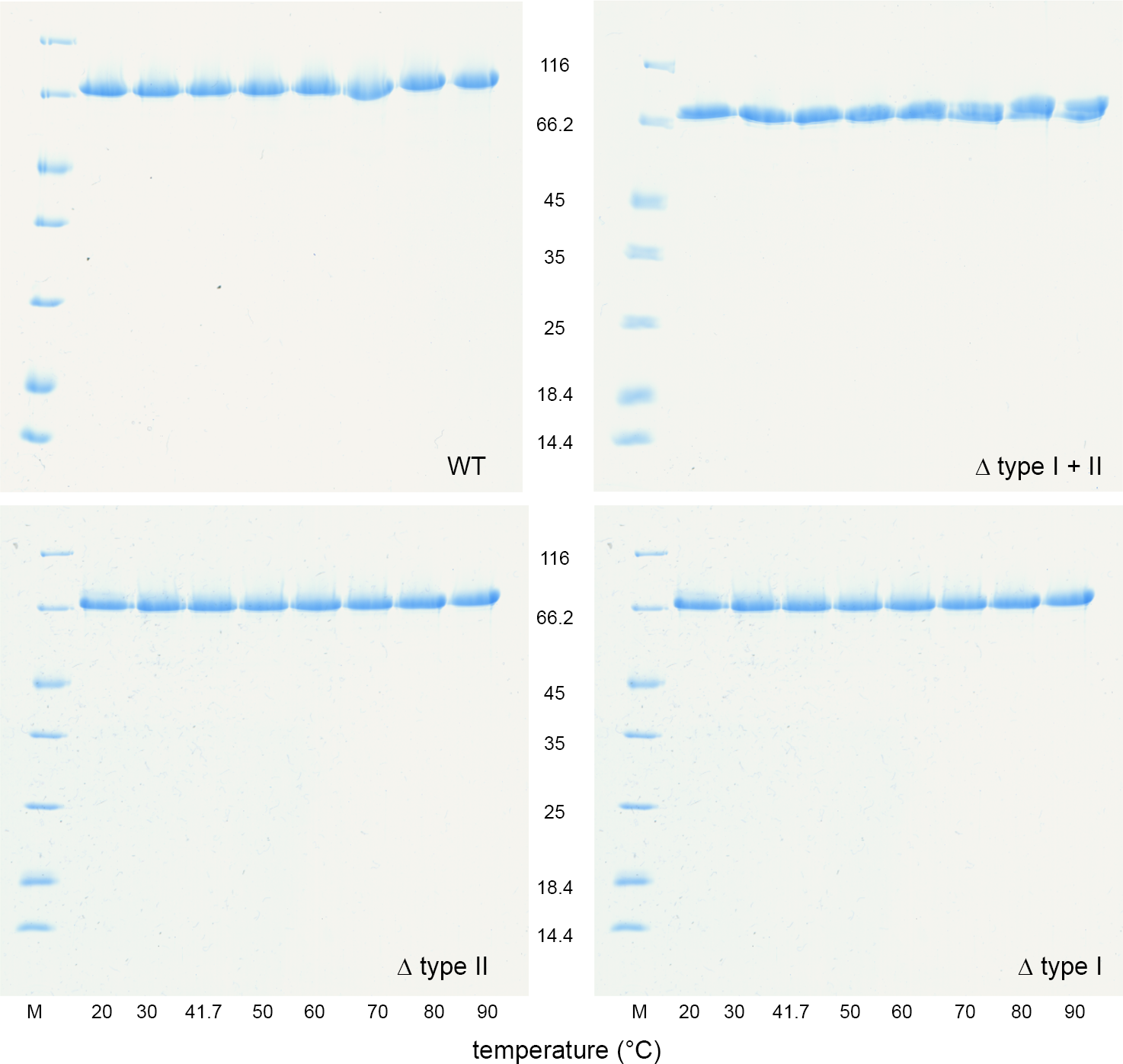

Supplement: S5 Fig — SiiECterm variants were subjected to incubation at various temperatures as indicated, and samples are analyzed SDS-PAGE rather than by native PAGE as for Fig 6E. The molecular weights of the marker bands (M) are indicated. (TIF) [file ppat.1006418.s009.tif]

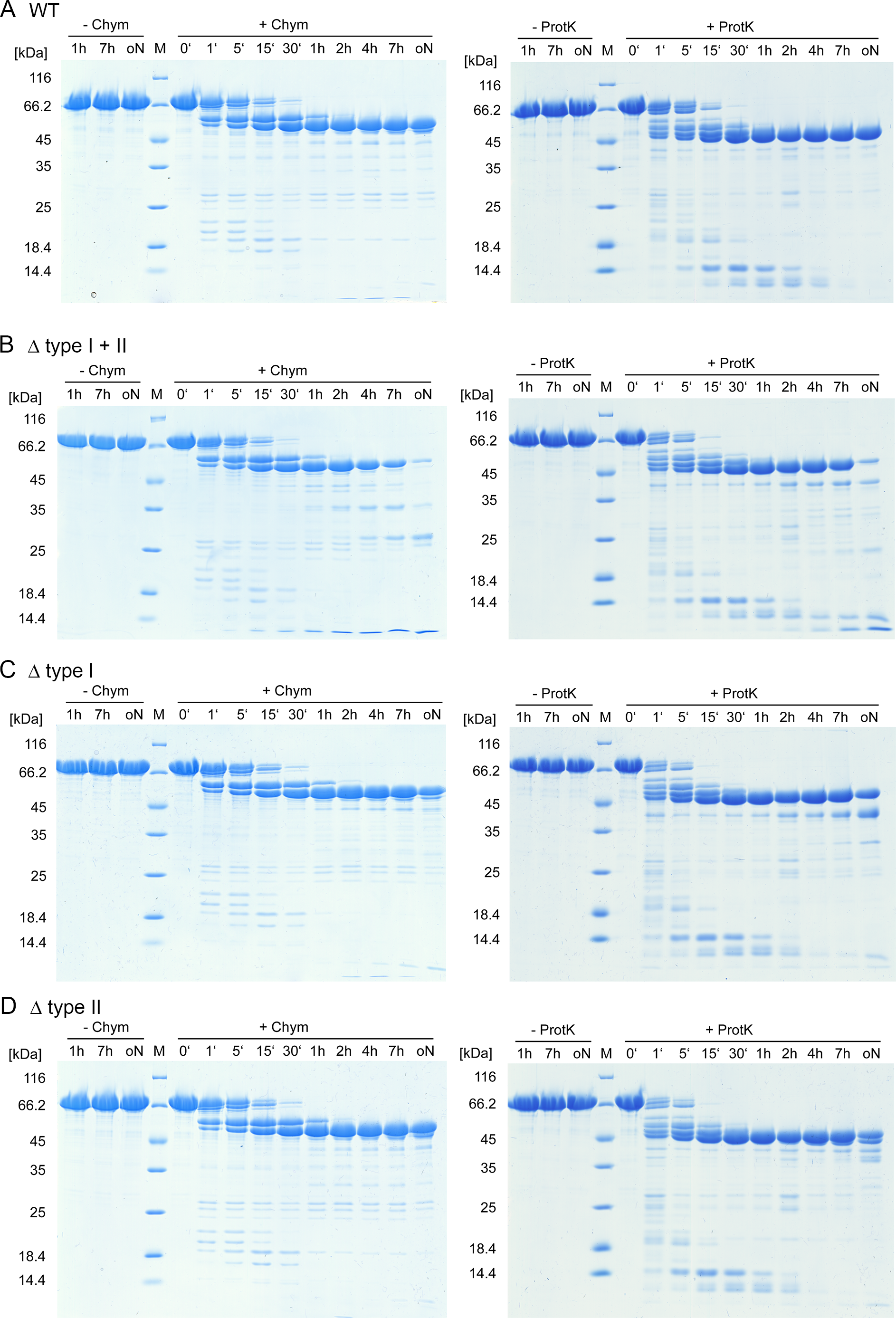

Supplement: S6 Fig — C-terminal portions of SiiE comprising BIg50 to the C-terminus (SiiECterm) were subjected to limited proteolysis by Chymotrypsin (Chym, left column) or Proteinase K (ProtK, right column). SiiECterm WT (WT, A), mutant proteins with D/S exchange in type I and type II Ca2+-binding sites (B), type I-binding sites only (C), or type II-binding sites only (D) were analyzed. Proteins were incubated with proteases for various time intervals as indicated (‘, minutes; h, hours; oN, overnight), reactions were stopped and degradation was analyzed by SDS-PAGE. Samples without protease (-ProtK, -Chym) were incubated and analyzed accordingly as negative controls. The molecular weights of the individual marker bands (M) are indicated. (TIF) [file ppat.1006418.s010.tif]

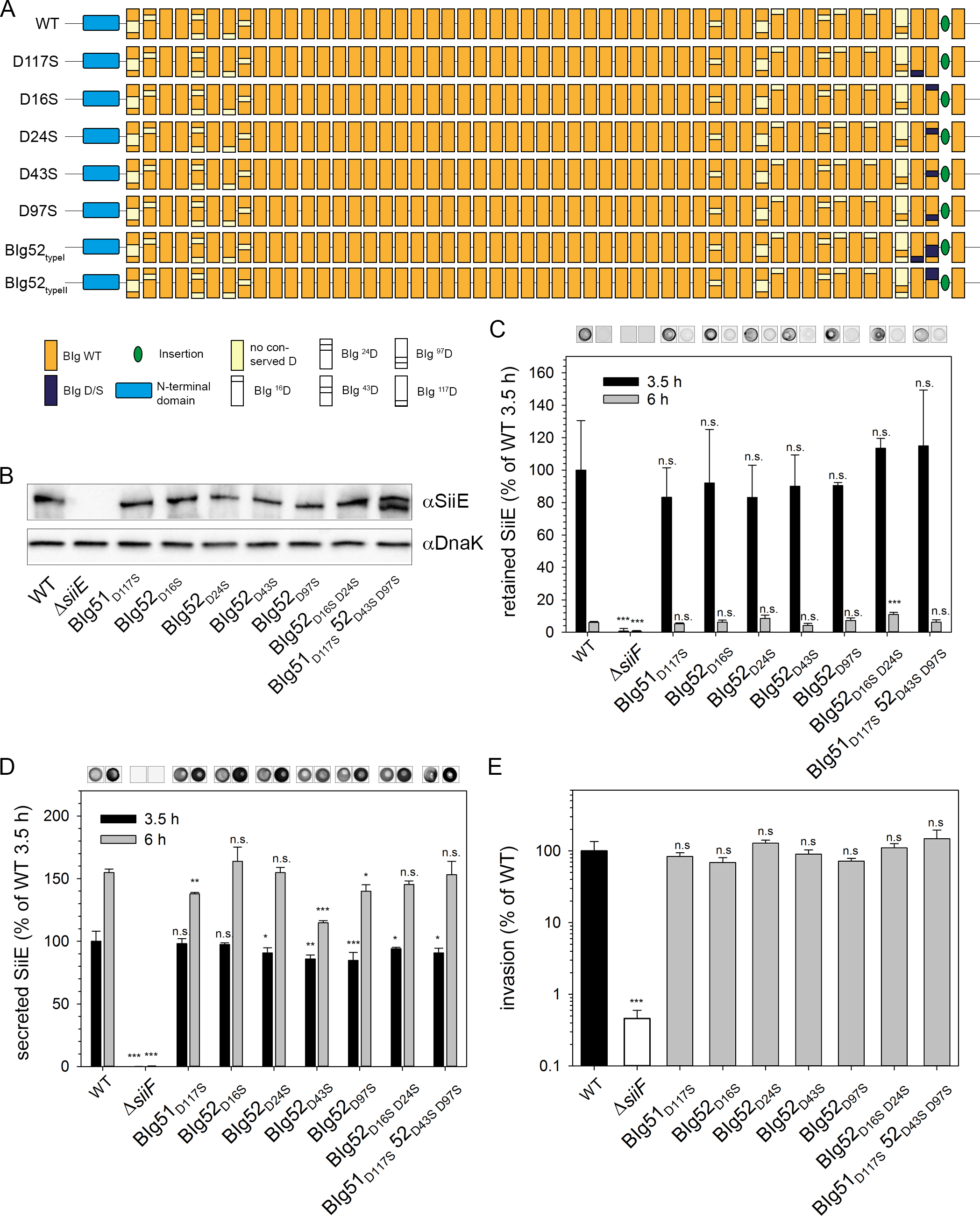

Supplement: S7 Fig — Mutations of chromosomal siiE were generated resulting in single D/S exchanges in BIg51/52, or D/S exchanges of type I or type II Ca2+-binding sites. A) Schematic overview of mutant siiE alleles. B) Synthesis of the mutant SiiE variants was tested by Western blot. C) Analyses of amounts of retained SiiE (C) and secreted SiiE (D) after 3.5 h and 6 h of subculture. E) SiiE-dependent invasion of polarized epithelial MDCK cells. Analyses of synthesis, surface retention and secretion and SiiE-dependent invasion of polarized cells were performed as described for Fig 2 of the main text. (TIF) [file ppat.1006418.s011.tif]
